# Missing data approaches for probability regression models with missing outcomes with applications

**DOI:** 10.1186/s40488-014-0023-3

**Published:** 2014-12-16

**Authors:** Li Qi, Yanqing Sun

**Keywords:** Augmented inverse probability weighted estimator, Asymptotic results, Automated records, Auxiliary outcome, Efficiency, Inverse probability weighted Estimator, Mean score estimation, Recurrent events, Vaccine efficacy, Validation sample

## Abstract

In this paper, we investigate several well known approaches for missing data and their relationships for the parametric probability regression model *P_β_*(*Y*|*X*) when outcome of interest *Y* is subject to missingness. We explore the relationships between the mean score method, the inverse probability weighting (IPW) method and the augmented inverse probability weighted (AIPW) method with some interesting findings. The asymptotic distributions of the IPW and AIPW estimators are derived and their efficiencies are compared. Our analysis details how efficiency may be gained from the AIPW estimator over the IPW estimator through estimation of validation probability and augmentation. We show that the AIPW estimator that is based on augmentation using the full set of observed variables is more efficient than the AIPW estimator that is based on augmentation using a subset of observed variables. The developed approaches are applied to Poisson regression model with missing outcomes based on auxiliary outcomes and a validated sample for true outcomes. We show that, by stratifying based on a set of discrete variables, the proposed statistical procedure can be formulated to analyze automated records that only contain summarized information at categorical levels. The proposed methods are applied to analyze influenza vaccine efficacy for an influenza vaccine study conducted in Temple-Belton, Texas during the 2000-2001 influenza season.

## 1 Introduction

Suppose that *Y* is the outcome of interest and *X* is a covariate vector. One is often interested in the probability regression model *P_β_*(*Y*|*X*) that relates *Y* to *X*. In many medical and epidemiological studies, the complete observations on *Y* may not be available for all study subjects because of time, cost, or ethical concerns. In some situations, an easily measured but less accurate outcome named auxiliary outcome variable, *A*, is supplemented. The relationship between the true outcome *Y* and the auxiliary outcome *A* in the available observations can inform about the missing values of *Y*. Let *V* be a subsample of the study subjects, termed the validation sample, for which both true and auxiliary outcomes are available. Thus observations on (*X*, *Y*, *A*) are available for the subjects in *V* and only (*X*, *A*) are observed for those not in *V*.

It is well known that the complete-case analysis, which uses only subjects who have all variables observed, can be biased and inefficient, cf. Little and Rubin (2002). The issues also rise when substituting auxiliary outcome for true outcome; see Ellenberg and Hamilton (1989), Prentice (1989) and Fleming (1992). Inverse probability weighting (IPW) is a statistical technique developed for surveys by Horvitz and Thompson (1952) to calculate statistics standardized to a population different from that in which the data was collected. This approach has been generalized to many aspects of statistics under various frameworks. In particular, the IPW approach is used to account for missing data through inflating the weight for subjects who are underrepresented due to missingness. The method can potentially reduce the bias of the complete-case estimator when weighting is correctly specified. However, this approach has been shown to be inefficient in several situations, see Clayton et al. (1998) and Scharfstein et al. (1999). Robins et al. (1994) developed an improved augmented inverse probability weighted (AIPW) complete-case estimation procedure. The method is more efficient and possesses double robustness property. The multiple imputation described in Rubin (1987) has been routinely used to handle missing data. Carpenter et al. (2006) compared the multiple imputation with IPW and AIPW, and found AIPW as an attractive alternative in terms of double robustness and efficiency. Using the maximum likelihood estimation (MLE) coupled with the EM-algorithm (Dempster et al. 1977), Pepe et al. (1994) proposed the mean score method for the regression model *P_β_* (*Y*|*X*) when both *X* and *A* are discrete.

In this paper, we investigate several well known approaches for missing data and their relationships for the parametric probability regression model *P_β_*(*Y*|*X*) when outcome of interest *Y* is subject to missingness. We explore the relationships between the mean score method, IPW and AIPW with some interesting findings. Our analysis details how efficiency is gained from the AIPW estimator over the IPW estimator through estimation of validation probability and augmentation to the IPW score function. Applying the developed missing data methods, we derive the estimation procedures for Poisson regression model with missing outcomes based on auxiliary outcomes and a validated sample for true outcomes. Further, we show that the proposed statistical procedures can be formulated to analyze automated records that only contain aggregated information at categorical levels, without using observations at individual levels.

The rest of the paper is organized as follows. Section 2 introduces several missing data approaches for the probability regression model *P_β_*(*Y*|*X*), where the outcome *Y* may be missing. Section 3 explores the relationships among these estimators. The asymptotic distributions of the IPW and AIPW estimators are derived and their efficiencies are compared. Section 3 investigates efficiency of two AIPW estimators, one is based on the augmentation using a subset of observed variables and the other is based on the augmentation using the full set of observed variables. The procedures for Poisson regression using automated data with missing outcomes are derived in Section 4. The finite-sample performances of the estimators are studied in simulations in Section 5. The proposed method is applied to analyze influenza vaccine efficacy for an influenza vaccine study conducted in Temple-Belton, Texas during the 2000-2001 influenza season. The proofs of the main results are given in the Appendix A, while the proof of a simplified variance formula in Section 4 is placed in the Appendix B.

## 2 Missing data approaches

Consider the probability regression model *P_β_*(*Y*|*X*), where *Y* is the outcome of interest and *X* is a covariate vector. Let *A* be the auxiliary outcome for *Y* and *V* be the validation set such that observations on (*X*, *Y*, *A*) are available for the subjects in *V* and only (*X*, *A*) are observed for those in *V̄*, the complement of *V*. In practice, the validation sample may be selected based on the characteristics of a subset, *Z*, of the covariates in *X*. We write *X* = (*Z*, *Z^c^*). For example, *Z* may include exposure indicator and other discrete covariates and *Z^c^* may be the exposure time. Let (*Z_i_*, *X_i_*, *Y_i_*, *A_i_*), *i* = 1, …, *n*, be independent identically distributed (iid) copies of (*Z*, *X*, *Y*, *A*). Let *ξ_i_* = *I*(*i* ∈ *V*) be the selection indicator.

Most statistical methods for missing data require some assumptions on missingness mechanisms. The commonly used ones are missing completely at random (MCAR) and missing at random (MAR). MCAR assumes that the probability of missingness in a variable is independent of any characteristics of the subjects. MAR assumes that the probability that a variable is missing depends only on observed variables. In practice, if missingness is a result by design, it is often convenient to let the missing probability depend on the categorical variables only. There is also simplicity in statistical inference by modeling the missing probability based on the categorical variables. We introduce the following missing at random assumptions.

MAR I: *ξ_i_* is independent of *Y_i_* conditional on (*X_i_*, *A_i_*) and *ξ_i_* is independent of $Z_{i}^{c}$ conditional on (*Z_i_*, *A_i_*).
MAR II: *ξ_i_* is independent of $\left(Y_{i},Z_{i}^{c}\right)$ conditional on (*Z_i_*, *A_i_*).

Since the conditional density *f*(*y*, *z^c^*|*ξ*, *z*, *a*) = *f*(*z^c^*|*ξ*, *z*, *a*)*f*(*y*|*z^c^*, *ξ*, *z*, *a*) = *f*(*z^c^*|*z*, *a*) *f*(*y*|*z^c^*, *z*, *a*) = *f*(*y*, *z^c^*|*z*, *a*), MAR I implies MAR II. It is also easy to show that MAR II implies MAR.

Let *π̂_i_* be the estimator of the conditional probability *π_i_* = *P*(*ξ_i_* = 1|*X_i_*, *A_i_*), and ${\hat{\pi }}_{i}^{z}$ the estimator of $\pi_{i}^{z}=P(\xi_{i}=1|Z_{i},A_{i})$. Let *S_β_*(*Y*|*X*) denote the partial derivatives of log *P_β_*(*Y*|*X*) with respect to *β*. Let *Ê*{*S_β_*(*Y* | *X_i_*)|*X_i_*, *A_i_*} be the estimator of the conditional expectation *E*{*S_β_*(*Y*|*X_i_*)|*X_i_*, *A_i_*}, and *Ê*{*S_β_*(*Y*|*X_i_*)|*Z_i_*, *A_i_*} the estimator of *E* {*S_β_*(*Y*|*X_i_*)|*Z_i_*, *A_i_*. We investigate several estimators of *β* based on the following estimating equations with different choices of *W_i_*:

(1) $$\sum_{i=1}^{n}W_{i}=0,$$

where *W_i_* takes one of the following forms:

(2) $$W_{i}^{I1}=\frac{\xi_{i}}{{\hat{\pi }}_{i}^{z}}S_{\beta }(Y_{i}|X_{i})$$

(3) $$W_{i}^{E1}=\xi_{i}S_{\beta }(Y_{i}|X_{i})+(1-\xi_{i})\hat{E}\{S_{\beta }(Y|X_{i})|Z_{i},A_{i}\}$$

(4) $$W_{i}^{A1}=\frac{\xi_{i}}{{\hat{\pi }}_{i}^{z}}S_{\beta }(Y_{i}|X_{i})+(1-\frac{\xi_{i}}{{\hat{\pi }}_{i}^{z}})\hat{E}\{S_{\beta }(Y|X_{i})|Z_{i},A_{i}\}$$

(5) $$W_{i}^{I2}=\frac{\xi_{i}}{{\hat{\pi }}_{i}}S_{\beta }(Y_{i}|X_{i})$$

(6) $$W_{i}^{E2}=\xi_{i}S_{\beta }(Y_{i}|X_{i})+(1-\xi_{i})\hat{E}\{S_{\beta }(Y|X_{i})|X_{i},A_{i}\}$$

(7) $$W_{i}^{A2}=\frac{\xi_{i}}{{\hat{\pi }}_{i}^{z}}S_{\beta }(Y_{i}|X_{i})+(1-\frac{\xi_{i}}{{\hat{\pi }}_{i}^{z}})\hat{E}\{S_{\beta }(Y|X_{i})|X_{i},A_{i}\}.$$

(8) $$W_{i}^{A3}=\frac{\xi_{i}}{{\hat{\pi }}_{i}}S_{\beta }(Y_{i}|X_{i})+(1-\frac{\xi_{i}}{{\hat{\pi }}_{i}})\hat{E}\{S_{\beta }(Y|X_{i})|X_{i},A_{i}\}.$$

The estimator *β̂_I_*_1_ obtained by using $W_{i}^{I1}$ is an IPW estimator where a subject's validation probability $\pi_{i}^{z}$ depends only on the category defined by (*Z_i_*, *A_i_*). Because $E\{{\left(\pi_{i}^{z}\right)}^{-1}\xi_{i}S_{\beta }(Y_{i}|X_{i})\}=E\{S_{\beta }(Y_{i}|X_{i})\}=0$, the estimator *β̂_I_*_1_ is approximately unbiased. The estimator *β̂_I_*_2_ obtained by using $W_{i}^{I2}$ is also an IPW estimator but with the validation probability *π_i_* depending on the category defined by (*Z_i_*, *A_i_*) and the additional covariate $Z_{i}^{c}$.

The estimator *β̂_E_*_1_ obtained by using $W_{i}^{E1}$ is the mean score estimator where the scores *S_β_*(*Y_i_*|*X_i_*) for those with missing outcomes are replaced by the estimated conditional expectations given (*Z_i_*, *A_i_*). The estimator *β̂_E_*_2_ obtained by using $W_{i}^{E2}$ is the mean score estimator where the scores *S_β_*(*Y_i_*|*X_i_*) for those with missing outcomes are replaced by the estimated conditional expectations given (*X_i_*, *A_i_*). The estimator *β̂_E_*_2_ is the mean score estimator in Pepe et al. (1994). The mean score estimator is the MLE estimator employing the EM-algorithm (Dempster et al. 1977) under the assumption that the auxiliary outcome is noninformative in the sense that the probability model *P_θ_* (*A* | *Y*, *X*) is unrelated to *β*.

The estimator *β̂_A_*_1_ obtained using $W_{i}^{A1}$ is the AIPW estimator augmented with the estimated conditional expectation *Ê* {*S_β_*(*Y*|*X_i_*)|*Z_i_*, *A_i_*}. The estimator *β̂_A_*_2_ obtained using $W_{i}^{A2}$ is the AIPW estimator augmented with the estimated conditional expectation *Ê*{*S_β_*(*Y*|*X_i_*)|*X_i_*, *A_i_*}. The estimator *β̂_A_*_3_ is obtained using $W_{i}^{A3}$. The $W_{i}^{A3}$ differs from $W_{i}^{A2}$ in that the estimated validation probability is *π̂_i_* instead of ${\hat{\pi }}_{i}^{z}$.

Suppose that ${\hat{\pi }}_{i}^{z}$ is an asymptotically unbiased estimator of ${\bar{\pi }}_{i}^{z}$ and that *Ê*{*S_β_*(*Y*|*X_i_*)|*Z_i_*, *A_i_*} is asymptotically unbiased of *Ē* {*S_β_*(*Y*|*X_i_*)|*Z_i_*, *A_i_*}, where both ${\bar{\pi }}_{i}^{z}$ and *Ē* {*S_β_*(*Y*|*X_i_*)|*Z_i_*, *A_i_*} are functions of (*Z_i_*, *A_i_*). Under MAR II, if one of the equalities, ${\bar{\pi }}_{i}^{z}=\pi_{i}^{z}$ and *Ē* [*S_β_*(*Y*|*X_i_*)|*Z_i_*, *A_i_*]} = *E*[*S_β_* (*Y*|*X_i_*)|*Z_i_*, *A_i_*]}, holds, then

$$E\{{\left({\bar{\pi }}_{i}^{z}\right)}^{-1}\xi_{i}S_{\beta }(Y_{i}|X_{i})\}+E\{(1-{\left(\pi_{i}^{z}\right)}^{-1}\xi_{i})\bar{E}[S_{\beta }(Y|X_{i})|Z_{i},A_{i}]\}=E\{S_{\beta }(Y_{i}|X_{i})\}=0,$$

which entails that the estimator *β̂_A_*_1_ has the double robust property in the sense that it is a consistent estimator of *β* if either ${\hat{\pi }}_{i}^{z}$ is a consistent estimator of $\pi_{i}^{z}$ or *Ê* [*S_β_* (*Y*|*X_i_*) |*Z_i_*, *A_i_*] is a consistent estimator of *E* [*S_β_*(*Y*|*X_i_*)|*Z_i_*, *A_i_*]. Similarly, under MAR I, the estimator *β̂_A_*_2_ possesses the double robust property in that *β̂_A_*_2_ is a consistent estimator of *β* if either ${\hat{\pi }}_{i}^{z}$ is a consistent estimator of $\pi_{i}^{z}$ or *Ê* [*S_β_* (*Y*|*X_i_*) |*X_i_*, *A_i_*] is a consistent estimator of *E* [*S_β_*(*Y*|*X_i_*)|*X_i_*, *A_i_*]. The estimator *β̂_A_*_3_ has similar double robust property as *β̂_A_*_2_.

## 3 Method comparisons and asymptotic results

Let *V*(*X_i_*, *A_i_*) denote the subjects in *V* with values of (*X*, *A*) equal to (*X_i_*, *A_i_*), *n^V^* (*X_i_*, *A_i_*) the number of subjects in *V*(*X_i_*, *A_i_*), and *n*(*X_i_*, *A_i_*) the number of subjects with values of (*X*, *A*) equal to (*X_i_*, *A_i_*). When *X* and *A* are discrete and their dimensionality is reasonably small, the probability *π_i_* = *P*(*ξ_i_* = 1|*X_i_*, *A_i_*) can be estimated by *π̂_i_* = *n^V^* (*X_i_*, *A_i_*)/*n*(*X_i_*, *A_i_*). The conditional expectation *E* {*S_β_*(*Y*|*X_i_*)|*X_i_*, *A_i_*} can be nonparametrically estimated based on the validation sample,

(9) $$\hat{E}\{S_{\beta }(Y|X_{i})|X_{i},A_{i}\}=\sum_{j\in V(X_{i},A_{i})}S_{\beta }(Y_{j}|X_{j})/n^{V}(X_{i},A_{i}),$$

Under MAR I, *Ê* {*S_β_* (*Y*|*X_i_*) |*X_i_*, *A_i_*} is an unbiased estimator of *E* {*S_β_* (*Y*|*X_i_*) |*X_i_*, *A_i_*}. Now we let *V*(*Z_i_*, *A_i_*) denote the subjects in *V* with values of (*Z*, *A*) equal to (*Z_i_*, *A_i_*), *n^V^* (*Z_i_*, *A_i_*) the number of subjects in *V*(*Z_i_*, *A_i_*), and *n*(*Z_i_*, *A_i_*) the number of subjects in the sample with values of (*Z*, *A*) equal to (*Z_i_*, *A_i_*). A nonparametric estimator of $\pi_{i}^{z}=P(\xi_{i}=1|Z_{i},A_{i})$ is given by ${\hat{\pi }}_{i}^{z}=n^{V}(Z_{i},A_{i})/n(Z_{i},A_{i})$. A nonparametric estimator of *E*{*S_β_*(*Y*|*X_i_*)|*Z_i_*, *A_i_*} is given by

(10) $$\hat{E}\{S_{\beta }(Y|X_{i})|Z_{i},A_{i}\}=\sum_{j\in V(Z_{i},A_{i})}S_{\beta }(Y_{j}|X_{j})/n^{V}(Z_{i},A_{i}).$$

Under MAR II, (*Y_i_*, *X_i_*) is independent of *ξ_i_* conditional on (*Z_i_*, *A_i_*), then *Ê*{*S_β_*(*Y*|*X_i_*)|*Z_i_*, *A_i_*} is an unbiased estimator of *E*{*S_β_*(*Y*|*X_i_*)|*Z_i_*, *A_i_*}.

**Proposition 1.** Suppose that *X* = (*Z*, *Z^c^*) and *A* are discrete and their dimensionality is reasonably small. Under the nonparametric estimators ${\hat{\pi }}_{i}^{z}=n^{V}(Z_{i},A_{i})/n(Z_{i},A_{i})$, *π̂_i_* = *n^V^* (*X_i_*, *A_i_*)/*n*(*X_i_*, *A_i_*) and the estimators for the conditional expectation defined in (9) and (10), the estimators *β̂_I_*_1_, *β̂_E_*_1_ and *β̂_A_*_1_ are equivalent, and the estimators *β̂_I_*_2_, *β̂_E_*_2_, *β̂_A_*_2_ and *β̂_A_*_3_ are equivalent. However, the estimator *β̂_A_*_2_ is different from *β̂_A_*_1_ unless $Z_{i}^{c}$ is linearly related to *Z_i_* in which case *β* is not identifiable.

The results of Proposition 1 are very intriguing since research has shown that the AIPW and the mean score methods are more efficient than the IPW method. It is also intriguing that the AIPW estimators *β̂_A_*_2_ and *β̂_A_*_3_ are actually the same estimators, not affected by the validation probability. To further understand these approaches, we investigate the asymptotic properties of these methods where (*X*, *A*) are not necessarily discrete. Through the asymptotic analysis, we gain insights about what matters to the efficiency in terms of the selections of the validation sample and the augmentation function.

Suppose that *Ẽ*{*S_β_* (*Y*|*X_i_*)|*X_i_*, *A_i_*} is a consistent parametric/nonparametric estimator of *E_a_* {*S_β_*(*Y*|*X_i_*)|*X_i_*, *A_i_*}, where *E_a_* {*S_β_*(*Y*|*X_i_*)|*X_i_*, *A_i_*} *is E* {*S_β_*(*Y*|*X_i_*)|*X_i_*, *A_i_*} or *E*{*S_β_*(*Y*| *X_i_*)|*Z_i_*, *A_i_*}. Let *π*(*X_i_*, *A_i_*, *ψ*) be the parametric model for the validation probability *π_i_*, where *ψ* is a *q*-dimensional parameter. We show in Corollary 2 that the nonparametric estimator of *π*(*X_i_*, *A_i_*, *ψ*) can also be expressed in the parametric form when (*X_i_*, *A_i_*) are discrete. Let *ψ*_0_ be the true value of *ψ*. Under MAR I, the MLE *ψ̂* = (*ψ̂*_1_, …, *ψ̂_q_*) of *ψ* = (*ψ*_1_, …, *ψ_q_*) is obtained by maximizing the observed data likelihood,

$$\prod_{i=1}^{n}\{\pi {\left(X_{i},A_{i},\psi )\right\}}^{\xi_{i}}{\left\{1-\pi (X_{i},A_{i},\psi )\right\}}^{1-\xi_{i}}.$$

The validation probability *π_i_* is estimated by *π̃_i_* = *π* (*X_i_*, *A_i_*, *ψ̂*). Then by the standard likelihood based analysis, we have the approximation

(11) $$\hat{\psi }-\psi_{0}=n^{-1}\sum_{i=1}^{n}{\left(I^{\psi }\right)}^{-1}S_{i}^{\psi }+o_{p}(n^{-1/2}),$$

where $S_{i}^{\psi }$ and *I^ѱ^* are the score vector and information matrix for *ѱ̂* defined by

(12) $$\begin{array}{c} S_{i}^{\psi }=\frac{\left(\xi_{i}-\pi (X_{i},A_{i},\psi_{0})\right)}{\pi (X_{i},A_{i},\psi_{0})(1-\pi (X_{i},A_{i},\psi_{0}))}\frac{\partial \pi (X_{i},A_{i},\psi_{0})}{\partial \psi },\\ I^{\psi }=E\{\frac{1}{\pi (X_{i},A_{i},\psi_{0})(1-\pi (X_{i},A_{i},\psi_{0}))}{\left(\frac{\partial \pi (X_{i},A_{i},\psi_{0})}{\partial \psi }\right)}^{⊗2}\}, \end{array}$$

where *a*^⊗2^ = *aa*′.

Consider the IPW estimator *β̂_I_* obtained by solving the estimating equation

(13) $$U_{I}=\sum_{i=1}^{n}\frac{\xi_{i}}{{\tilde{\pi }}_{i}}S_{\beta }(Y_{i}|X_{i})$$

and the AIPW estimator *β̂_A_* based on solving the estimating equation

(14) $$U_{A}=\sum_{i=1}^{n}[\frac{\xi_{i}}{{\tilde{\pi }}_{i}}S_{\beta }(Y_{i}|X_{i})+(1-\frac{\xi_{i}}{{\tilde{\pi }}_{i}})\tilde{E}\{S_{\beta }(Y|X_{i})|X_{i},A_{i}\}].$$

**Theorem 1.** Assume that *P_β_*(*Y*|*X*) and *π*(*X*, *A*, *ѱ*) have bounded third-order derivatives in a neighborhood of the true parameters and are bounded away from 0 almost surely, both −*E* {(∂^2^/∂*β*^2^) (log *P_β_*(*Y*|*X*))} and *I^ѱ^* are positive definite at the true parameters. Then, under MAR I,

$$\begin{array}{c} n^{1/2}({\hat{\beta }}_{I}-\beta )=I^{-1}(\beta )n^{-1/2}\sum_{i=1}^{n}Q_{i}^{I}+o_{p}(1),\\ n^{1/2}({\hat{\beta }}_{A}-\beta )=I^{-1}(\beta )n^{-1/2}\sum_{i=1}^{n}Q_{i}^{A}+o_{p}(1), \end{array}$$

where *I*(*β*) = *E* {−(∂^2^/∂*β*^2^) (log *P_β_*(*Y*|*X*))} = *Var*(*S_β_* (*Y_i_*|*X_i_*)),

$$Q_{i}^{I}=\xi_{i}/\pi_{i}S_{\beta }(Y_{i}|X_{i})-E\{\pi_{i}^{-2}\xi_{i}S_{\beta }(Y_{i}|X_{i}){\left(\partial \pi (X_{i},A_{i},\psi_{0})/\partial \psi \right)}^{\prime }\}{\left(I^{\psi }\right)}^{-1}S_{i}^{\psi }$$

and $Q_{i}^{A}=\xi_{i}/\pi_{i}S_{\beta }(Y_{i}|X_{i})+(1-\xi_{i}/\pi_{i})E_{a}\{S_{\beta }(Y|X_{i})|X_{i},A_{i}\}$.

Both *n*^1/2^ (*β̂_I_* − *β*) and *n*^1/2^ (*β̂_A_* − *β*) have asymptotically normal distributions with mean zero and covariances equal to $I^{-1}(\beta )\text{Var}(Q_{i}^{I})I^{-1}(\beta )$ and $I^{-1}(\beta )\text{Var}(Q_{I}^{A})I^{-1}(\beta )$, respectively. Further,

(15) $$\text{Var}(Q_{i}^{I})=\text{Var}(Q_{i}^{A})+\text{Var}(B_{i}+O_{i})$$

and

(16) $$\text{Var}(Q_{i}^{A})=I(\beta )+\text{Var}((1-\frac{\xi_{i}}{\pi_{i}})\{S_{\beta }(Y_{i}|X_{i})-E_{a}\{S_{\beta }(Y|X_{i})|X_{i},A_{i}\}\}),$$

where $O_{i}=E\{\pi_{i}^{-2}\xi_{i}S_{\beta }(Y_{i}|X_{i}){\left(\partial \pi (X_{i},A_{i},\psi_{0})/\partial \psi \right)}^{\prime }\}{\left(I^{\psi }\right)}^{-1}S_{i}^{\psi }$ and *B_i_* = (1 − *ξ_i_*/*π_i_*) *E_a_* {*S_β_* (*Y*|*X_i_*) |*X_i_*, *A_i_*}.

Suppose that the validation probability *π_i_* = *P*(*ξ_i_*=1|*X_i_*, *A_i_*) depends only on (*Z_i_*, *A_i_*). That is, $\pi_{i}=\pi_{i}^{z}=P(\xi_{i}=1|Z_{i},A_{i})$. Suppose that *π̃_i_* is the MLE of $\pi_{i}^{z}$ under the parametric family *ѱ* = (*Z_i_*, *A_i_*, *ѱ*). Let *β̂_A_*_1_ be the estimator obtained by solving (14) where the augmented term, *Ẽ*{*S_β_* (*Y*|*X_i_*)|*X_i_*, *A_i_*}, is a consistent parametric/nonparametric estimator of *E* {*S_β_*(*Y*|*X_i_*)|*Z_i_*, *A_i_*. Let *β̂_A_*_2_ be the estimator obtained by solving (14) where *Ẽ* {*S_β_*(*Y*|*X_i_*)|*X_i_*, *A_i_*} is a consistent parametric/nonparametric estimator of *E* {*S_β_*(*Y*|*X_i_*)|*X_i_*, *A_i_*}. The following corollary presents the asymptotic results for two AIPW estimators of *β*, one that corresponds to the augmentation based on a subset, (*Z*, *A*), of observed variables and the other that corresponds to the augmentation based on the full set, (*X*, *A*), of the observed variables.

**Corollary 1.** Suppose that the validation probability *π_i_* = *P* (*ξ_i_* = 1 |*X_i_*, *A_i_*) depends only on (*Z_i_*, *A_i_*). Under the conditions of Theorem 1,

(17) $$n^{1/2}({\hat{\beta }}_{A1}-\beta )\overset{D}{\to }N(0,I^{-1}(\beta )+I^{-1}(\beta )\sum_{A1}(\beta )I^{-1}(\beta )),$$

and

(18) $$n^{1/2}({\hat{\beta }}_{A2}-\beta )\overset{D}{\to }N(0,I^{-1}(\beta )+I^{-1}(\beta )\sum_{A2}(\beta )I^{-1}(\beta )),$$

where $\sum_{A1}(\beta )=E[((1-\pi_{i}^{z})/\pi_{i}^{z})\text{Var}\{S_{\beta }(Y_{i}|X_{i})|Z_{i},A_{i}\}]$ and $\sum_{A2}(\beta )=E[((1-\pi_{i}^{z})/\pi_{i}^{z})\text{Var}\{S_{\beta }(Y_{i}|X_{i})|X_{i},A_{i}\}]$. The asymptotic variance of *β̂_A_*_2_ is smaller than the asymptotic variance of *β̂_A_*_1_ if the covariates *Z_i_* are a proper subset of *X_i_*.

Suppose that (*Z*, *A*) are discrete taking values (*z*, *a*) in a set *Ƶ* of finite number of values. If the number of parameters in *ѱ* equals the number of values *ѱ*_*z*, *a*_ = *P*(*ξ_i_* = 1 |*Z_i_* =*z*, *A_i_* = *a*) for all distinct pairs (*z*, *a*), then *ѱ* = {*ѱ*_*z*, *a*_} and *n*(*z*, *a*, *ѱ*) = *ѱ*_*z*, *a*_. Further, $\frac{\partial \pi (z,a,\psi_{0})}{\partial \psi }$ can be viewed as a column vector with 1 in the position for *ѱ*_*z*, *a*_ and 0 elsewhere. The information matrix *I^ѱ^* defined in (12) has the expression,

$$I^{\psi }=\sum_{z,a}\rho (z,a)\{\frac{1}{\pi (z,a,\psi_{0})(1-\pi (z,a,\psi_{0}))}\frac{\partial \pi (z,a,\psi_{0})}{\partial \psi }{\left(\frac{\partial \pi (z,a,\psi_{0})}{\partial \psi }\right)}^{\prime }\},$$

where *ρ*(*z*, *a*) = *P*(*Z_i_* = *z*, *A_i_* = *a*). *It* follows that *I^ѱ^* is a diagonal matrix and its inverse matrix is also diagonal. The MLE *ѱ̂*_*z*, *a*_ = *n^V^* (*z*, *a*)/*n*(*z*, *a*) is in fact the nonparametric estimator for *ѱ*_*z*, *a*_ based on the proportion of validated samples in the category specified by (*z*, *a*). The equation (11) can be expressed as

$${\hat{\psi }}_{z,a}-\pi (z,a,\psi_{0})=n^{-1}\frac{n^{V}(z,a)-n(z,a)\pi (z,a,\psi_{0})}{\rho (z,a)}+o_{p}(n^{-1/2}),$$

for (*z*, *a*) ∈ *Ƶ*.

By Threom 1, the possible efficiency gain of the AIPW estimator over the IPW estimator is shown through the equation (15). The AIPW estimator is more efficient unless Var(*B_i_* + *O_i_*) = 0. In particular, from the proof of Theorem 1, we have

(19) $$n^{-1/2}U_{A}=n^{-1/2}\sum_{i=1}^{n}Q_{i}^{A}+o_{p}(1)$$

(20) $$n^{-1/2}U_{I}=n^{-1/2}\sum_{i=1}^{n}Q_{i}^{A}-n^{-1/2}\sum_{i=1}^{n}(B_{i}+O_{i})+o_{p}(1),$$

where *B_i_* and *O_i_* are defined following (16). The following corollary presents the analysis of the term $n^{-1/2}\sum_{i=1}^{n}(B_{i}+O_{i})$ when (*Z_i_*, *A_i_*) are discrete to understand how efficiency may be gained from the AIPW estimator over the IPW estimator.

**Corollary 2.** Under the conditions of Theorem 1, suppose that *X* = (*Z*, *Z^c^*) and (*Z*, *A*) are discrete taking values (*z*, *a*) in a set *Ƶ* of finite number of values. Suppose that the validation probability *π_i_* = *P*(*ξ_i_* = 1|*X_i_*, *A_i_*) only depends on (*Z_i_*, *A_i_*) and *ѱ*_*z*, *a*_ = *P*(*ξ_i_* = 1|*Z_i_* = *z*, *A_i_* = *a*) is estimated nonparametrically by *ѱ̂*_*z*, *a*_ = *n^V^*(*z*, *a*)/*n*(*z*, *a*). Then

(21) $$n^{-1/2}\sum_{i=1}^{n}(O_{i}+B_{i})=-\sum_{z,a}n^{-1/2}\sum_{j=1}^{n}\frac{\xi_{j}-\pi (z,a,\psi_{0})}{\pi (z,a,\psi_{0})}I(Z_{j}=z,A_{j}=a)[E\{S_{\beta }(Y_{j}|X_{j})|Z_{j}=z,A_{j}=a\}-E\{S_{\beta }(Y_{j}|X_{j})|Z_{j}=z,Z_{j}^{c},A_{j}=a\}].$$

By Corollary 2, (19) and (20), *β̂_A_* is more efficient than *β̂_I_* unless *Var*{*S_β_*(*Y_j_*|*X_j_*)|*Z_j_* = *z*, *A_j_* = *a*} = 0 for all (*z*, *a*) for which *P*(*Z_i_* = *z*, *A_i_* = *a*) *≠* 0. If *X* = *Z* and the validation probability *π_i_* = *P*(*ξ_i_* = 1 |*X_i_*, *A_i_*) is nonparametrically estimated with the cell frequencies *ѱ̂_z, a_* = *n^V^* (*z*, *A*)/*n*(*z*, *a*), then *β̂_a_* and *β̂_I_* are asymptotically equivalent.

**Remark** Consider the estimators of *β* obtained based on the estimating equation (1) corresponding to different choices of *W_i_* given in (2) to (8). If (*Z*, *A*) are discrete and the validation probability $\pi_{i}^{z}=P(\xi_{i}=1|Z_{i},A_{i})$ is estimated nonparametrically by the cell frequency, then by Theorem 1 and Corollary 2, *β̂_A_*_1_ and *β̂_I_*_1_ have same asymptotic normal distributions as long as *Ê*[*S_β_*(*Y*|*X_i_*)|*Z_i_*, *A_i_*] is a consistent estimator of *E*[*S_β_*(*Y*|*X_i_*)|*Z_i_*, *A_i_*]. But *β̂_A_*_2_ is more efficient than *β̂_I_*_1_ as long as *Ê*[*S_β_*(*Y*|*X_i_*)|*X_i_*, *A_i_*] is a consistent estimator of *E*[*S_β_*(*Y*|*X_i_*)|*X_i_*, *A_i_*] since Var(*B_i_* + *O_i_*) is not zero by (21). These results are not affected by whether *E*[*S_β_*(*Y*|*X_i_*)|*Z_i_*, *A_i_*] and *E*[*S_β_*(*Y*|*X_i_*)|*X_i_*, *A_i_*] are estimated nonparametrically or based on some parametric models. In addition, by Theorem 1, Corollary 1 and 2, *β̂*_A3_ and *β̂_I_*_2_ have the same asymptotic normal distributions as long as *Ê*[*S_β_*(*Y*|*X_i_*)|*X_i_*, *A_i_*] is a consistent estimator of *E*[*S_β_*(*Y*|*X_i_*)|*X_i_*, *A_i_*].

## 4 Poisson regression using the automated data with missing outcomes

Many medical and public health data are available only in aggregated format, where the variables of interest are aggregated counts without being available at individual levels. Many existing statistical methods for missing data require observations at individual levels. Applying the missing data methods presented in Section 3, we derive some estimation procedures for the Poisson regression model with missing outcomes based on auxiliary outcomes and a validated sample for true outcomes. Further, we show that, by stratifying based on a set of discrete variables, the proposed statistical procedure can be formulated so that it can be used to analyze automated records which do not contain observations at individual levels, only summarized information at categorical levels.

Let *Y* represent the number of events occurring in the time-exposure interval [0, *T*] and *Z* the covariates. We consider the Poisson regression model,

(22) $$P(Y=y|Z,T)=\exp\{-T\exp(\beta^{\prime }Z)\}{\left\{T\exp(\beta^{\prime }Z)\right\}}^{y}/y!,$$

where *Z* is a vector of *k* + 1 covariates and *β* a vector of *k* + 1 regression coefficients. In practice, the exact number of true events may not be available for all subjects. We may instead have the number of possible events, namely, the auxiliary events. For example, in the study of vaccine adverse events associated with childhood immunizations, the number of auxiliary events *A* for MAARI is collected based on ICD-9 codes through hospital records. Further diagnosis may indicate that some of these events are false events. The number of true vaccine adverse events, *Y*, can only be confirmed for the subjects in the validation set *V*. Suppose that *Z* is the vaccination status, 1 for the vaccinated and 0 for the unvaccinated. Then, under Poisson regression, exp(*β*) is the relative rate of event occurrence per unit time of the exposed versus unexposed. We assume that the number of automated events *A* can be expressed as *A* = *Y* + *W*, where *W* is the number of false events independent of *Y* conditional on (*Z*, *T*). Suppose that *W* follows the Poisson regression model

(23) $$P(W=w|Z,T)=\exp\{-T\exp(\gamma^{\prime }Z)\}{\left\{T\exp(\gamma^{\prime }Z)\right\}}^{w}/w!,$$

where *γ*′ = (*a*_0_, *a*_1_, *γ*_1_, ⋯, *γ_k_*_−1_).

We apply the missing data methods introduced in Section 3 on model (22). The variables (*Z_i_*, *T_i_*, *Y_i_*, *A_i_*) are observed for the validation sample *V* and (*Z_i_*, *T_i_*, *A_i_*) are observed for the nonvalidation sample *V̄*. While the covariate *Z* can be considered as categorical, it is natural to consider the exposure time *T* as a continuous variable. We assume that the validation probability depends only on the stratification of (*Z*, *A*). That is, the validation sample is a stratified random sample by the categories defined by (*Z*, *A*). Of those estimators discussed in Section 2, there are only two different estimators, *β̂_I_*_1_ and *β̂_A_*_2_. We show in Section 4.3 that the proposed method can be formulated so that it can be used to analyze the automated records with missing outcomes. First we derive the explicit estimation procedures for *β̂_I_*_1_ and *β̂_A_*_2_ and their variance estimators under model (22).

### 4.1 Inverse probability weighting estimation

We adopt all notations introduced in Section 3. In particular, let $\pi_{i}^{z}=P(\xi_{i}=1|Z_{i},A_{i})$ and ${\hat{\pi }}_{i}^{z}=n^{V}(Z_{i},A_{i})/n(Z_{i},A_{i})$. Let *X* = (*Z*, *T*) and *X_i_* = (*Z_i_*, *T_i_*) to be consistent with earlier notations. The score function for subject *i* under model (22) is $S_{\beta }(Y_{i}|X_{i})=Z_{i}^{\prime }(Y_{i}-T_{i}\exp(\beta^{\prime }Z_{i}))$. The estimator *β̂_I_*_1_ is obtained by solving $\sum_{i=1}^{n}(\xi_{i}/{\hat{\pi }}_{i}^{z})S_{\beta }(Y_{i}|X_{i})=0$, where $S_{\beta }(Y_{i}|X_{i})=Z_{i}^{\prime }(Y_{i}-T_{i}\exp(\gamma^{\prime }Z_{i}))$. By Corollary 1, √*n*(*β̂_I_*_1_ − *β*) converges in distribution to a normal distribution with mean zero and the variance matrix *I*^−1^(*β*) + *I*^−1^ (*β*)Σ*_A_*_1_(*β*)*I*^−1^ (*β*), where $\sum_{A1}(\beta )=E[((1-\pi_{i}^{z})/\pi_{i}^{z})\text{Var}\{S_{\beta }(Y_{i}|X_{i})|Z_{i},A_{i}\}]$.

The information matrix $I(\beta )=E(Z_{i}Z_{i}^{\prime }T_{i}\exp(\beta^{\prime }Z_{i}))=\sum_{z}P(Z_{i}=z)zz^{\prime }\exp(\beta^{\prime }z)E(T_{i}|Z_{i}=z)$ can be estimated by *Î*(*β̂*) which is obtained by replacing *β* with *β̂_I_*_1_, *P*(*Z_i_* = *z*) by the sample proportion of the event {*Z_i_* = *z*}, and *E*(*T_i_*|*Z_i_* = *z*) with the sample average exposure time for those with covariates *Z_i_* = *z*. The matrix Σ*_A_*_1_(*β*) can be estimated by

(24) $${\hat{\sum }}_{A1}(\hat{\beta })=\sum_{a,z}\hat{\rho }(a,z)\frac{1-{\hat{\rho }}^{v}(a,z)}{{\hat{\rho }}^{v}(a,z)}\hat{\text{Var}}\{S_{\beta }(Y|X)|A=a,Z=z\},$$

where *ρ̂*(*a*, *z*) is the estimator of *P*{*A_i_* = *a*, *Z_i_* = *z*}, *ρ̂^v^*(*a*, *z*) is the estimator of *P*{*i* ∈ *V*|*A_i_* = *a*, *Z_i_* = *z*}, and $\hat{\text{Var}}\{S_{\beta }(Y|X)A=a,Z=z\}$ is an estimator of Var {*S_β_* (*Y_i_*|*X_i_*)|*Z_i_*, *A_i_*}] which is derived in the following.

Since *A* is observed for all subjects, *W* can be determined if *Y* is known, and undetermined otherwise. The IPW estimator, *γ̂_I_*_1_, of *γ* can be estimated by solving the equation $\sum_{i=1}^{n}(\xi_{i}/{\hat{\pi }}_{i}^{z})S_{\gamma }(W_{i}|X_{i})=0$, where $S_{\gamma }(W_{i}|X_{i})=Z_{i}^{\prime }(W_{i}-T_{i}\exp(\gamma^{\prime }Z_{i}))$. The conditional distribution of *Y* given *A* = *a*, *T*, and *Z* = *z* is Binomial (*a*, *p_z_*), where *p_z_* = exp(*β*′*z*)/(exp(*β*′*z*) + exp(*γ*′*z*)). Since this conditional distribution does not depend on *T*, the outcome *Y* and *T* are conditionally independent given (*A*, *Z*). Therefore, Var {*S_β_*(*Y* |*X*)|*A*, *Z*} = *ZZ*' {Var(*Y*|*A*, *Z*) + exp (2*β*′*Z*)Var(*T*|*A*, *Z*)}. The variance Var(*Y*|*A* = *a*, *Z* = *z*) can be estimated by *ap̂_z_*(1 − *p̂_z_*), where *p̂_z_* = exp(*β̂*′*z*)/{exp(*β̂*′*z*) + exp(*γ̂*′*z*)}, and Var(*T*|*A* = *a*, *Z* = *z*) = *E*(*T^2^*|*A* = *a*, *Z* = *z*) − {*E*(*T*|*A* = *a*, *Z* = *z*)}*^2^* can be estimated nonparametrically using the first and the second sample moments conditional on each category with *A* = *a* and *Z* = *z*.

### 4.2 Augmented inverse probability weighted estimation

Under the assumption that *W* follows the Poisson regression model (23) and is independent of *Y* conditional on (*Z*, *T*), $E\{S_{\beta }(Y|X)Z,T,A\}=AZ^{\prime }\frac{\exp(\beta^{\prime }Z)}{\exp(\beta^{\prime }Z)+\exp(\gamma^{\prime }Z)}-TZ^{\prime }\exp(\beta^{\prime }Z)$. Let *Ê* {*S_β_*(*Y*|*X_i_*)|*X_i_*, *A_i_*} be the estimator of *E* {*S_β_*(*Y* |*X_i_*)|*X_i_*, *A_i_*} for a given *β* by substituting *γ* by its estimator *γ̂_I_*_1_ of Section 4.1. Then the estimator *β̂_A_*_2_ is obtained by solving

(25) $$\sum_{i=1}^{n}\frac{\xi_{i}}{{\hat{\pi }}_{i}^{z}}S_{\beta }(Y_{i}|X_{i})+(1-\frac{\xi_{i}}{{\hat{\pi }}_{i}^{z}})\hat{E}\{S_{\beta }(Y|X_{i})|X_{i},A_{i}\}=0.$$

By Corollary 1, √*n*(*β̂_A_*_2_ − *β*) converges in distribution to a normal distribution with mean zero and the variance matrix where *I*^−1^ (*β*) +*I*^−1^ (*β*) Σ*_A_*_2_ (*β*)*I*^−1^ (*β*), where $\sum_{A2}(\beta )=E[((1-\pi_{i}^{z})/\pi_{i}^{z})\text{Var}\{S_{\beta }(Y_{i}|X_{i})|X_{i},A_{i}\}]$. The information matrix *I*(*β*) can be estimated by *Î*(*β̂*) given in Section 4.1. The conditional variance Var {*S_β_*(*Y* |*X*)|*Z* = *z*, *T*, *A* = *a*} = *ap_z_*(1 − *p_z_*)*z*^⊗2^ can be estimated by *ap̂_z_*(1 − *p̂_z_*), where *p̂_z_* = exp(*β̂*′*_z_*)/(exp(*β̂*′*z*) + exp(*γ̂*′*z*)). It follows that Σ*_A_*_2_(*β*) can be consistently estimated by

$${\hat{\sum }}_{A2}(\beta )=\sum_{a,z}\hat{\rho }(a,z)\frac{1-{\hat{\rho }}^{v}(a,z)}{{\hat{\rho }}^{v}(a,z)}a{\hat{p}}_{z}(1-{\hat{p}}_{z})z^{⊗2},$$

where *ρ̂*(*a*, *z*) is the estimator of *P*{*A_i_* = *a*, *Z_i_* = *z*} and *ρ̂^v^*(*a*, *z*) is the estimator of *P*{*i* ∈ *V*|*A_i_* = *a*, *Z_i_* = *z*}.

### 4.3 Estimation using the automated data

This section formulates the missing data estimation procedure for (22) based on the automated (summarized) information at categorical levels defined by relevant covariates of the model. In particular, we show that *β̂_I_*_1_ and *β̂_A_*_2_ and their variance estimators can be formulated using the automated data at categorical levels.

In many applications it is convenient to write *Z* = (1, *Z*_(1)_, *Z*_(2)_) and *β* = (*b_0_*, *b*_1_, *θ*′)′, where *Z*_(1)_ is the treatment indicator (*Z*_(1)_ = 1 for the exposed group and *Z*_(1)_ = 0 for the unexposed group) and *Z*_(2)_ = (*η*_1_, ⋯, *η_k_*_−1_)′ as the other covariates, and *θ* = (*θ*_1_, ⋯, *θ_k_*_−1_)′. For the applications involving the automated data records, we let *η*_1_, ⋯, *η_k_*_−1_ be *k* − 1 dummy variables representing *k* groups. Without loss of generality, we choose the *k*th group as the reference group, *η*_1_ = 1, *η*_2_ = 0, ⋯, *η_k_*_−1_ = 0 for group 1, *η*_1_ = 0, *η*_2_ = 1, ⋯, *η_k_*_−1_ = 0 for group 2, so on and *η*_1_ = 0, *η*_2_ = 0, ⋯, *η_k_*_−1_ = 0 for group *k*. Thus each value of *Z* denotes a category which can be represented by (*l*, *m*) for *l* = 0, 1 and *m* = 1, ⋯, *k*. This correspondence is denoted by *Z* ⋍ (*l*, *m*) for convenience. For *l* = 0, 1 and *m* = 1, ⋯, *k* − 1, category (*l*, *m*) is defined by *Z* with *Z*_(1)_ = *l*, *η_m_* = 1 and *η_j_* = 0 for *j* ≠ *m*, *m*, *j*= 1, …, *k*, and category (*l*, *k*) is defined by *Z*_(1)_ = *l* and *η_j_* = 0 for *j* = 1, …, *k* − 1. Under model (22), the expected number of events for a subject in category (*l*, *m*) with the time-exposure interval [0, *T*] is *T* exp(*b_lm_*), for *l* = 0, 1 and *m* = 1, ⋯, *k*, where *b*_1_*_k_* = *b*_0_ + *b*_1_, *b*_0_*_k_* = *b*_0_, *b*_1_*_m_* = *b*_0_ + *b*_1_+*θ_m_* and *b*_0_*_m_* = *b*_0_ + *θ_m_* for 1 ≤ *m* ≤ *k* − 1. The parameter *b*_1_ represents the log-relative rate of the exposed versus the unexposed adjusted for other factors.

The following notations are used to show that the estimators of *β* and their variance estimators can be calculated using the automated information at the categorical levels. Let *V*(*a*, *l*, *m*) denote the set of subjects in *V* with (*A* = *a*, *Z* ⋍ (*l*, *m*)), *V*(*l*, *m*) for the set of subjects in *V* with (*Z* ⋍ (*l*, *m*)), *n_alm_* for the number of subjects with (*A* = *a*, *Z* ⋍ (*l*, *m*)), $n_{\text{alm}}^{v}$ for the number of subjects in *V*(*a*, *l*, *m*), $n_{\text{lm}}^{v}$ for the number of subjects in $V(l,m),\lambda_{\text{alm}}=n_{\text{alm}}/n_{\text{alm}}^{v}$, *y_alm_* for the number of events for subjects in *V*(*a*, *l*, *m*), *y_lm_* for the number of events for subjects in *V*(*l*, *m*), *t_alm_* for the total exposure time for subjects with (*A* = *a*, *Z* ⋍ (*l*, *m*)), *t*_2,_ *_alm_* for the total squared exposure time for subjects with (*A* = *a*, *Z* ⋍ (*l*, *m*)), *t_lm_* for the total exposure time for subjects with *Z* ⋍ (*l*, *m*), *α_lm_* for the number of automated events for subjects with *Z* ⋍ (*l*, *m*).

#### Estimation with *β̂_I_*_1_ using the automated data

The validation probability $\pi_{i}^{z}$ can be estimated by 1/*λ_alm_* when *A_i_* = *a*, *Z_i_* ⋍ (*l*, *m*). It can be shown that the estimating equation for *β̂_I_*_1_ is equivalent to the following nonlinear equations for {*b_lm_*, for *l* = 0, 1, *m* = 1, ⋯, *k*},

$$\begin{array}{c} \sum_{m=1}^{k}(\sum_{a\in A}y_{\text{alm}}\lambda_{\text{alm}}-e^{b_{lm}}\sum_{a\in A}t_{\text{alm}}\lambda_{\text{alm}})=0,\\ \sum_{l=0,1}(\sum_{a\in A}y_{\text{alm}}\lambda_{\text{alm}}-e^{b_{lm}}\sum_{a\in A}t_{\text{alm}}\lambda_{\text{alm}})=0, \end{array}$$

for *l* = 0, 1 and *m* = 1, …, *k* − 1. When *k >* 1, the equations have no explicit solutions.

In the following, we show that the asymptotic variance of *β̂_I_*_1_ can be consistently estimated by only using the automated information at categorical levels. The information matrix is a **(***k* + 1) × **(***k* + 1**)** symmetric matrix given by

$$I(\beta )=E(Z_{i}Z_{i}^{\prime }T_{i}\exp(\beta^{\prime }Z_{i}))=[\begin{array}{ccccc} \sum_{l,m}q_{lm} & \sum_{m=1}^{k}q_{1m} & q_{11}+q_{01} & \cdots & q_{1r}+q_{0r}\\ \sum_{m=1}^{k}q_{1m} & \sum_{m=1}^{k}q_{1m} & q_{11} & \cdots & q_{1r}\\ q_{11}+q_{01} & q_{11} & q_{11}+q_{01} & \cdots & 0\\ \vdots & \vdots & \vdots & \ddots & \vdots \\ q_{1r}+q_{0r} & q_{1r} & 0 & \cdots & q_{1r}+q_{0r} \end{array}],$$

where *r* = *k* − 1 and *q_lm_* = *E*(*T_i_e ^blm^I*{individual *i* in category (*l*, *m*)}). The consistent estimator, *Î*(*β̂*), of *I*(*β*) is thus obtained by replacing *q_lm_* with *exp*(*b̂_lm_*)*t_lm_*/*n*.

Under model (23), the expected number of false events for a subject in category (*l*, *m*) with the time-exposure interval [0, *T*] is *T* exp(*d_lm_*), for *l* = 0, 1 and *m* = 1, ⋯, *k*, where *d*_1_*_k_* = *a*_0_ + *a*_1_, *d*_0_*_k_* = *a*_0_, *d*_1_*_m_* = *a*_0_ + *a*_1_ + *γ_m_* and *d*_0_*_m_* = *a*_0_ + *γ_m_* for 1 ≤ *m* ≤ *k* − 1. The conditional distribution of *Y* given *A* = *a*, *T*, and *Z* ⋍ (*l*, *m*) is Binomial (*a*, *p_lm_*), where *p_lm_* = exp(*b_lm_*)/(exp(*b_lm_*) + exp(*d_lm_*)) for *a* ≥ 1. Then Var(*Y*|*A* = *a*, *Z* ⋍ (*l*, *m*)) can be estimated by *ap̂_lm_*(1 −*p_lm_*), where *p_lm_* = *e^b̂_lm_^*/(*e^b̂_lm_^* + *e^d̂_lm_^*), and Var(*T*|*A* = *a*, *Z* ⋍ (*l*, *m*)) can be estimated by *ν*_*a*, *l*, *m*_ = *t*_2, *a*, *l*, *m*_/*n_alm_* − (*t_alm_*/*n_alm_*)^2^. By (24) and the discussion that follows, Σ*_A_*_1_(*β*) can be estimated by

(26) $${\hat{\sum }}_{A1}(\hat{\beta })=\sum_{a,l,m}\hat{\rho }(a,l,m)\frac{1-{\hat{\rho }}^{v}(a,l,m)}{{\hat{\rho }}^{v}(a,l,m)}G_{lm}\{a{\hat{p}}_{lm}(1-{\hat{p}}_{lm})+v_{a,l,m}\},$$

where $\hat{\rho }(a,l,m)=n_{\text{alm}}/n,{\hat{\rho }}^{v}(a,l,m)=n_{\text{alm}}^{v}/n_{\text{alm}}$ and *G_lm_* be the value of $G_{i}=z_{i}^{⊗2}$ when subject *i* belongs to the category (*l*, *m*). Hence the covariance matrix of *β̂_I_*_1_ can be estimated by *Î*^−1^ (*β̂*) + *Î*^−1^(*β̂*) Σ*^_A_*_1_ (*β̂*)*I*^−1^ (*β̂*) using the automated data.

#### Estimation with *β̂_A_*_2_ using the automated data

The estimating equations (25) are equivalent to the following nonlinear equations for {*b_lm_*, for *l* = 0, 1, *m* = 1, ⋯, *k*},

$$\begin{array}{c} \sum_{m=1}^{k}\{\sum_{a\in A}y_{\text{alm}}\lambda_{\text{alm}}-e^{b_{lm}}t_{lm}+\frac{e^{b_{lm}}}{e^{b_{lm}}+e^{{\hat{d}}_{1m}}}(\alpha_{lm}-\sum_{a\in A}an_{\text{alm}}\lambda_{\text{alm}})\}=0,\\ \sum_{l=0,1}\{\sum_{a\in A}y_{\text{alm}}\lambda_{\text{alm}}-e^{b_{lm}}t_{lm}+\frac{e^{b_{lm}}}{e^{b_{lm}}+e^{{\hat{d}}_{lm}}}(\alpha_{lm}-\sum_{a\in A}an_{\text{alm}}\lambda_{\text{alm}})\}=0, \end{array}$$

for *l* = 0, 1 and *m* = 1, …, *k* − 1.

Since Var {*S_β_*(*Y*|*X*)|*Z* ⋍ (*l*, *m*), *T*, *A* = *a*} = *ap_lm_*(1 − *p_lm_*)*G*_*lm*_, Σ*_A_*_2_(*β*) can be consistently estimated by

$${\hat{\sum }}_{A2}(\hat{\beta })=\sum_{a,l,m}\hat{\rho }(a,l,m)\frac{1-{\hat{\rho }}^{v}(a,l,m)}{{\hat{\rho }}^{v}(a,l,m)}a{\hat{p}}_{lm}(1-{\hat{p}}_{lm})G_{lm}.$$

Hence the covariance matrix of *β̂_A_*_2_ can be estimated by *Î*^−1^ (*β̂*) +*I*^−1^ (*β̂*)Σ*^_A_*_2_(*β̂*)*I*^−1^ (*β̂*) using the automated data.

**Remark** In the special case where *ρ*(*α*, *l*, *m*) *≈* 0 for *α ≥* 2, a much simpler formula for the variance estimator of the log relative risk can be derived. For example in the vaccine safety study, the adverse-event rate is very small. Let

$$w_{m}=\frac{\alpha_{0m}\alpha_{1m}y_{0m}y_{1m}}{\alpha_{0m}y_{0m}n_{1m}^{v}+\alpha_{1m}y_{1m}n_{0m}^{v}}/\sum_{m=1}^{k}\frac{\alpha_{0m}\alpha_{1m}y_{0m}n_{1m}^{v}}{\alpha_{0m}y_{0m}n_{1m}^{v}+\alpha_{1m}y_{1m}n_{0m}^{v}}.$$

Then an estimate of variance of *b̂*_1_ is given by

(29) $$\hat{\text{Var}({\hat{b}}_{1})}=\sum_{m=1}^{k}w_{m}^{2}(\frac{1}{y_{1m}}-\frac{1}{n_{1m}^{v}}+\frac{1}{\alpha_{1m}}+\frac{1}{y_{0m}}-\frac{1}{n_{0m}^{v}}+\frac{1}{\alpha_{0m}}),$$

which is the weighted sum of the estimated variances for the estimated log relative rate of the exposed versus the unexposed over *k* groups. The details of deviation are given in the Appendix B.

## 5 A simulation study

We conduct a simulation study to examine the finite sample performance of the IPW estimator *β̂_I_*_1_ and the AIPW estimator *β̂_A_*_2_. We consider the Poisson regression model (22). The covariates *Z*_1_ and *Z*_2_ are generated from the Bernoulli distributions with the probability of success equals to 0.4 and 0.5 respectively. The exposure time *T* is generated from a uniform distribution on [0, 10]. Given *Z* = (*Z*_1_, *Z*_2_) and *T*, the outcome variable *Y* follows a Poisson distribution with mean *T* exp (*b*_0_ + *b*_1_*Z*_1_ + *θZ*_2_) where *b*_0_ = −0.5, *b*_1_ = −0.8 and *θ* = −0.6, and *W* follows a Poisson distribution with mean *T* exp (*a*_0_ + *a*_1_*Z*_1_ + *γZ*_2_) where *a*_0_ = −1.3, *a*_1_ = −1.1, *γ* = −1. We set *A* = *Y* + *W*.

Four models for the validation sample are considered. Under Model 1, the validation sample is a simple random sample with probability *π_i_* = 0.4. Model 2 considers *π_i_* = 0.6. In Model 3, the validation probability only depends on *A* through the logistic regression model logit{*π_i_*(*X*, *A*)} = *A* − 0.5 where *X* = (*Z*, *T*). In Model 4, the validation probability depends on *A* and *Z*_1_ through the logistic regression model logit{*π_i_*(*X*, *A*)} = *A* − *Z*_1_ − 0.5.

Tables 1 and 2 present the simulation results for *n* = 50, 100, 300, 500 and 800. Each entry of the tables is based on 1000 simulation runs. Tables 1 and 2 summarize the bias (Bias), the empirical standard error (SSE), the average of the estimated standard error (ESE), and the empirical coverage probability (CP) of 95% confidence intervals of *β̂_I_*_1_ and *β̂_A_*_2_ for *β* = (*b*_0_, *b*_1_, *θ*). We also compare the performance of the estimators *β̂_I_*_1_ and *β̂_A_*_2_ with the complete-case (CC) estimator *β̂_C_* obtained by simply deleting subjects with missing values of *Y_i_*. As a gold standard, we present the estimation results for the full data where all the values of *Y_i_* are fully observed. Table 1 presents the results under Model 1 and 2, and Table 2 shows the results under Model 3 and 4.

Table 1 shows that under Model 1 and Model 2, the bias of all estimators is very small at a level comparable with that of the full data estimator. The bias decreases with increased sample size and the increased level of the validation probability. The empirical standard errors are in good agreement with the corresponding estimated standard errors, except for the IPW estimator when *n* ≤ 100 and *π* ≤ 0.6. Among them, AIPW has the smallest standard errors for all parameters and sample sizes concerned. The coverage probabilities of the confidence intervals for *b*_0_, *b*_1_ and *θ* are close to the nominal level 95%. When the sample size and the validation probability are both small, for example, *n* = 50 and *π* = 0.4, the IPW has large bias and is unstable but the AIPW still performs well.

Table 2 gives the results under Model 3 and Model 4. The bias remains small for *β̂_I_*_1_ and *β̂_A_*_2_. The empirical standard errors are also close to the corresponding estimated standard errors. The coverage probabilities remain close to the nominal level 95% for all IPW and AIPW estimators. However, the complete-case estimator yields larger bias and incorrect coverage probability because of the association between the validation probability and the auxiliary variable *A* and/or the covariate *Z*_1_, in which case the missing is not missing completely at random. The AIPW performs better than IPW with smaller standard errors.

## 6 An Application

A community-based, nonrandomized, open-label influenza vaccine (CAIV-T) study was conducted in Temple-Belton, Texas during the 2000-2001 influenza season. The total 11, 606 healthy children aged 18 months - 18 years were involved in this study and about 20% of them received a single dose of CAIV-T in 2000. The primary clinical outcome was based on an nonspecific case definition called medically attended acute respiratory infection (MAARI), which included all International Classification of Diseases, Ninth Revision, Clinical Modification diagnoses codes (ICD-9 codes 381-383, 460-487) for upper and lower respiratory tract infections, otitis media and sinusitis. MAARI outcomes and demographic data were extracted from the Scott & White Health Plan administrative database. For each visit, one or two International Classification of Diseases, Ninth Revision, Clinical Modification diagnosis codes were listed. Visits for which asthma diagnosis codes alone were noted, without another MAARI code, were excluded. More details about this study can be found in Halloran et al. (2003).

Any children representing with history of fever and any respiratory illness were eligible to have a throat swab for influenza virus culture. The decision to obtain specimens was made irrespective of whether a patient had received CAIV-T. The specific case definition was culture-confirmed influenza. Table 3 taken from Halloran et al. (2003) contains information on the number of children in three age groups, the number of children who are vaccinated versus unvaccinated, the number of nonspecific MAARI cases, the number of cultures performed, and the number of cultures positive for each group.

With the method developed in Section 4 for Poisson regression, we compare the risk of developing MAARI for children who received CAIV-T to the risk for children who had never received CAIV-T using the automated information provided in Table 3. The number of nonspecific MAARI cases extracted using the ICD-9 codes is the auxiliary outcome *A*, whereas the actual number of influenza cases *Y* is the outcome of interest. Let *Z*_1_ be the treatment indicator (1=vaccine and 0=placebo). Let *Z*_2_ = (*η*_1_, *η*_2_) be the dummy variables indicating three age groups, where *η*_1_ = 1 if the age is in the range 1.5– 4, *η*_1_ = 0, otherwise, and *η*_2_ = 1 if the age is in the range 5–9, *η*_2_ = 0, otherwise. The reference group is the age 10–18. The exposure time for all children is taken as *T* = 1 year.

Consider a Poisson regression model with mean *T* exp(*b*_0_ + *b*_1_*Z*_1_ + *θ*_1_*η*_1_ + *θ*_2_*η*_2_). Using the IPW estimator *β̂_I_*_1_, the estimates (standard errors) are *b̂*_0_ = −0.7659 (*σ̂_b_*_0_ = 0.1046), *b̂*_1_ = −1.5830 (*σ̂_b_*_1_ = 0.5017), *θ̂*_1_ = −0.5572 (*σ̂*_*θ*_1__ = 0.2111) and *θ̂*_2_ = −0.0199 (*σ̂*_*θ*_2__ = 0.1472). The age-adjusted relative rate (RR) in the vaccinated group compared with the unvaccinated group equals exp(*b̂*_1_) = exp(−1.5830) = 0.2054, which means that the rate of developing MAARI for the vaccinated group is 20% of that for the unvaccinated group. In terms of the vaccine efficacy VE = 1 − RR = 0.7946, this represents about 80% reduction in the risk of developing MAARI for the vaccinated group compared to the unvaccinated group. The 95% confidence interval of RR obtained by using the delta method is (0.0768, 0.5490), showing clear evidence that the vaccinated children have less risk of influenza than the unvaccinated children. The 95% confidence interval for VE is (0.4510, 0.9232).

Using the AIPW estimator *β̂_A_*_2_, the estimates (standard errors) are *b̂*_0_ = −2.0703 (*σ̂*_*b*_0__ = 0.0851), *b̂*_1_ = −1.8072(*σ̂*_*b*_1__ = 0.3786), *θ̂*_1_ = 0.6452 (*σ̂*_*θ*_1__ = 0.1966) and *θ̂*_2_ = 0.6235 (*σ̂*_*θ*_2__ = 0.1265). The age-adjusted relative rate (RR) is exp(*b̂*_1_) = exp(−1.8072) = 0.1641. The estimated VE is 0.8359 and the 95% confidence interval is (0.6553, 0.9219). The estimator *β̂_A_*_2_ yields smaller standard errors and confidence intervals with more precision than using *β̂_I_*_1_.

This data was analyzed by Halloran et al. (2003) and Chu and Halloran (2004). Assuming the binary probability model for *P_β_* (*Y*|*X*) where *X* includes the vaccination status and age group indicators, and using the mean score method, Halloran et al. (2003) found that the estimated VE based on the nonspecific MAARI cases alone was 0.18 with 95% confidence interval of (0.11, 0.24). The estimated VE by incorporating the surveillance cultures was 0.79 with 95% confidence interval of (0.51, 0.91). Halloran et al. also reported sample-size-weighted VE= 0.77 with 95% confidence interval of (0.48, 0.90). Chu and Halloran (2004) have developed a Bayesian method to estimate vaccine efficacy. By Chu and Halloran (2004), the estimated VE was 0.74 with 95% confidence interval (0.50, 0.88) and estimated VE by the multiple imputation method was 0.71 with 95% confidence interval (0.42, 0.86).

Our estimates of the vaccine efficacy are in line with the existing methods. The estimator *β̂_A_*_2_ yields smaller standard errors and therefore confidence intervals are more precise than the existing methods of Halloran et al. (2003) and Chu and Halloran (2004). Compared to the binary regression, Poisson regression model allows multiple recurrent MAARI cases for each child. Although for this particular application the exposure time is fixed at one year time interval, the proposed method is applicable to the situation where the length of exposure time may be different for different children.

## 7 Conclusions

In this paper, we investigated the mean score method, the IPW method and the AIPW method for the parametric probability regression model *P_β_*(*Y*|*X*) when outcome of interest *Y* is subject to missingness. The asymptotic distributions are derived for the IPW estimator and the AIPW estimator. The selection probability often needs to be estimated for the IPW estimator, and both the selection probability and the conditional expectation of the score function needs to be estimated for the AIPW estimator. We investigated the properties of the IPW estimator and the AIPW estimator when the selection probability and the conditional expectation are implemented differently.

An AIPW estimator is said to be fully augmented if the selection probability and the conditional expectation are estimated using the full set of observed variables; it is partially augmented if the selection probability and the conditional expectation are estimated using a subset of observed variables. Corollary 1 shows that the fully augmented AIPW estimator is more efficient than the partially augmented AIPW estimator. Corollary 2 shows that the AIPW estimator is more efficient than the IPW estimator. However, when the selection probability depends only on a set of discrete random variables, the IPW estimator obtained by estimating the selection probability nonparametrically with the cell frequencies is asymptotically equivalent to the AIPW estimator augmented using the same set of discrete random variables. Proposition 1 shows that the IPW estimator, the AIPW estimator and the mean score estimator are equivalent if the selection probability and the conditional expectation are estimated using same set of discrete random variables.

Applying the developed missing data methods, we derived the estimation procedures for Poisson regression model with missing outcomes based on auxiliary outcomes and a validated sample for true outcomes. By assuming the selection probability depending only on the observed discrete exposure variables, not on the continuous exposure time, we show that the IPW estimator and the AIPW estimator can be formulated to analyze data when only aggregated/summarized information are available. The simulation study shows that for a moderate sample size and selection probability, the IPW estimator and AIPW estimator perform better than the complete-case estimator. The AIPW estimator is more efficient and more stable than the IPW estimator. The proposed methods are applied to analyze a data set from for an influenza vaccine study conducted in Temple-Belton, Texas during the 2000-2001 influenza season. The data set presented in Table 3 only contains summarized information at categorical levels defined by the three age groups and vaccination status. The actual number of influenza cases (the number of positive cultures) out of the number of MAARI cases cultured, along with the number of MAARI cases, are available for each category. Our analysis using the AIPW approach shows that the age-adjusted relative rate in the vaccinated group compared to the unvaccinated group equals 0.1641, which represents about 84% reduction in the risk of developing MAARI for the vaccinated group compared to the unvaccinated group.

## Figures and Tables

**Table 1:** Simulation comparison of the IPW estimator *β̂_I_*_1_, the AIPW estimator *β̂_A_*_2_ and the complete-case (CC) estimator *β̂_C_* under various sample sizes and selection probabilities

		*b*_0_				*b*_1_				*θ*			
				
n		Bias	SSE	ESE	CP	Bias	SSE	ESE	CP	Bias	SSE	ESE	CP
**Model 1: *π_i_* = .4**													
50	IPW	-.0415	.3561	.1839	.851	-.2175	1.6737	.3354	.864	-.1610	1.2201	.2962	.847
	AIPW	-.0110	.2213	.1664	.890	-.0062	.3099	.2873	.943	-.0186	.3076	.2551	.929
	CC	-.0246	.3398	.2738	.938	-.1515	1.6082	.4730	.968	-.1038	1.1709	.4187	.959
100	IPW	-.0650	.1815	.1404	.870	-.0548	.3120	.2458	.891	-.0249	.2653	.2161	.898
	AIPW	-.0094	.1376	.1187	.914	-.0024	.2284	.1988	.926	.0027	.1994	.1780	.925
	CC	-.0240	.1728	.1685	.948	-.0086	.3086	.2981	.960	.0031	.2556	.2581	.946
300	IPW	-.0368	.0936	.0874	.931	-.0209	.1535	.1460	.946	-.0022	.1419	.1286	.929
	AIPW	-.0027	.0732	.0712	.946	-.0028	.1233	.1165	.940	.0005	.1130	.1046	.938
	CC	-.0092	.0919	.0935	.960	-.0012	.1627	.1634	.952	.0040	.1438	.1432	.952
500	IPW	-.0183	.0698	.0671	.938	-.0172	.1159	.1128	.943	-.0083	.1069	.0993	.933
	AIPW	.0022	.0566	.0550	.936	-.0022	.0956	.0902	.943	-.0068	.0867	.0811	.930
	CC	.0006	.0704	.0716	.949	-.0059	.1268	.1255	.949	-.0046	.1135	.1103	.942
800	IPW	-.0126	.0538	.0527	.942	-.0134	.0862	.0889	.950	-.0029	.0759	.0779	.947
	AIPW	.0011	.0433	.0435	.952	-.0047	.0720	.0713	.956	-.0020	.0638	.0640	.951
	CC	.0002	.0562	.0565	.948	-.0051	.0974	.0990	.958	-.0013	.0844	.0869	.958
**Model 2: *π_i_* = .6**													
50	IPW	-.0316	.2079	.1714	.926	-.0934	.8426	.3112	.944	-.0563	.3320	.2690	.937
	AIPW	-.0072	.1723	.1591	.941	-.0105	.2893	.2772	.950	-.0172	.2653	.2440	.948
	CC	-.0126	.1973	.1949	.959	-.0594	.8369	.3512	.967	-.0278	.3213	.3044	.959
100	IPW	-.0366	.1399	.1259	.926	-.0420	.2363	.2192	.944	-.0100	.2103	.1911	.925
	AIPW	-.0121	.1206	.1133	.941	-.0107	.2069	.1921	.944	.0078	.1764	.1700	.940
	CC	-.0142	.1370	.1345	.947	-.0216	.2379	.2370	.961	.0030	.2103	.2072	.949
300	IPW	-.0138	.0742	.0728	.944	-.0194	.1267	.1250	.957	-.0049	.1064	.1096	.964
	AIPW	-.0030	.0650	.0651	.948	-.0044	.1136	.1093	.949	.0005	.0960	.0974	.956
	CC	-.0017	.0763	.0759	.946	-.0118	.1345	.1328	.951	-.0035	.1147	.1169	.957
500	IPW	-.0069	.0571	.0555	.945	-.0096	.0946	.0965	.947	-.0094	.0866	.0844	.953
	AIPW	.0029	.0495	.0496	.942	-.0032	.0856	.0841	.947	-.0076	.0757	.0749	.955
	CC	.0013	.0577	.0581	.947	-.0034	.1024	.1019	.949	-.0086	.0906	.0899	.954
800	IPW	-.0072	.0437	.0438	.954	-.0069	.0754	.0763	.956	-.0025	.0692	.0664	.947
	AIPW	-.0011	.0401	.0393	.951	-.0019	.0693	.0665	.943	-.0015	.0626	.0590	.931
	CC	-.0012	.0452	.0460	.958	-.0026	.0805	.0806	.952	-.0024	.0723	.0709	.952
**Full data: *π_i_* = 1**													
50		-.0079	.1510	.1466	.952	-.0182	.2691	.2618	.948	-.0104	.2264	.2263	.957
100		-.0079	.1068	.1024	.943	-.0075	.1841	.1798	.948	-.0039	.1560	.1577	.949
300		-.0019	.0596	.0583	.950	-.0081	.1032	.1023	.936	.0001	.0934	.0898	.932
500		.0006	.0452	.0450	.951	-.0041	.0783	.0788	.950	.0014	.0656	.0693	.960
800		-.0004	.0343	.0356	.951	.0025	.0612	.0622	.938	-.0006	.0532	.0547	.955

**Table 2:** Simulation comparison of the IPW estimator *β̂_I_*_1_, the AIPW estimator *β̂_A_*_2_ and the complete-case (CC) estimator *β̂_C_* under various sample sizes and selection probabilities

		*b*_0_				*b*_1_				*θ*			
				
n		Bias	SSE	ESE	CP	Bias	SSE	ESE	CP	Bias	SSE	ESE	CP
**Model 3**													
50	IPW	.0081	.1609	.1502	.949	-.0034	.5535	.2790	.954	-.0116	.2592	.2400	.963
	AIPW	-.0070	.1543	.1486	.950	-.0134	.2715	.2690	.958	-.0185	.2364	.2330	.955
	CC	.0230	.1529	.1504	.938	.0798	.5441	.2835	.940	.0648	.2367	.2414	.952
100	IPW	-.0052	.1145	.1077	.948	-.0001	.2073	.2014	.959	.0030	.1789	.1724	.948
	AIPW	-.0124	.1077	.1041	.947	-.0085	.1869	.1840	.957	.0050	.1636	.1606	.948
	CC	.0221	.1074	.1054	.939	.1023	.1830	.1937	.924	.0828	.1625	.1664	.915
300	IPW	-.0011	.0617	.0614	.951	-.0044	.1176	.1157	.952	.0019	.1009	.0993	.953
	AIPW	-.0023	.0582	.0588	.956	-.0051	.1056	.1036	.954	.0022	.0936	.0910	.944
	CC	.0295	.0577	.0596	.924	.1051	.1070	.1095	.824	.0823	.0925	.0946	.861
500	IPW	.0018	.0451	.0473	.958	-.0037	.0853	.0895	.958	-.0069	.0765	.0767	.945
	AIPW	.0009	.0430	.0452	.957	-.0032	.0793	.0798	.947	-.0066	.0689	.0702	.951
	CC	.0317	.0429	.0459	.903	.1077	.0788	.0844	.763	.0737	.0704	.0730	.839
800	IPW	-.0006	.0374	.0375	.951	-.0030	.0671	.0708	.962	.0004	.0617	.0605	.946
	AIPW	-.0003	.0362	.0358	.949	-.0031	.0623	.0631	.954	-.0012	.0577	.0554	.935
	CC	.0315	.0353	.0364	.863	.1065	.0630	.0667	.633	.0786	.0568	.0576	.721
**Model 4**													
50	IPW	.0053	.1627	.1504	.948	.0825	.3531	.2832	.913	-.0057	.2736	.2405	.948
	AIPW	-.0085	.1549	.1489	.950	-.0122	.2746	.2752	.966	-.0138	.2395	.2340	.961
	CC	.2295	.2640	.0855	.531	.4513	.3805	.1760	.517	.2954	.3285	.1409	.536
100	IPW	-.0050	.1168	.1085	.939	.0481	.2350	.2130	.922	.0016	.1884	.1761	.940
	AIPW	-.0130	.1083	.1043	.943	-.0067	.1920	.1885	.950	.0066	.1648	.1613	.949
	CC	.0196	.1077	.1063	.943	.2010	.1946	.2087	.820	.0900	.1645	.1702	.910
300	IPW	-.0001	.0630	.0624	.945	-.0001	.1323	.1311	.955	-.0011	.1043	.1038	.946
	AIPW	-.0020	.0588	.0588	.951	-.0052	.1095	.1059	.952	.0012	.0950	.0913	.931
	CC	.0271	.0582	.0601	.930	.2020	.1060	.1173	.576	.0894	.0939	.0965	.863
500	IPW	.0012	.0457	.0480	.951	-.0007	.0966	.1010	.966	-.0054	.0801	.0799	.948
	AIPW	.0006	.0433	.0453	.956	-.0010	.0821	.0813	.941	-.0059	.0697	.0704	.950
	CC	.0291	.0434	.0463	.912	.2047	.0817	.0903	.364	.0815	.0711	.0745	.820
800	IPW	-.0006	.0381	.0380	.949	.0004	.0761	.0794	.967	.0000	.0636	.0630	.947
	AIPW	-.0002	.0362	.0359	.949	-.0016	.0640	.0641	.955	-.0014	.0574	.0555	.942
	CC	.0288	.0356	.0367	.885	.2039	.0644	.0714	.166	.0864	.0570	.0588	.673

**Table 3:** Study data for influenza epidemic season 2000-01, by age and vaccine group (from Halloran et al. 2003)

Age group (years)	Vaccine	No. of children	No. of MAARI cases	No. of MAARI cases cultured	No. of positive cultures
1.5-4	CAIV-T	537	389	16	0
	None	1844	1665	86	24
5-9	CAIV-T	807	316	17	2
	None	2232	1156	118	53
10-18	CAIV-T	937	219	19	3
	None	5249	1421	123	56

Total	CAIV-T	2281	924	52	5
	None	9325	4242	327	133

## Appendix A

### Proof of Proposition 1

Since

$$\sum_{i=1}^{n}(1-\xi_{i})\hat{E}\{S_{\beta }(Y|X_{i})|Z_{i},A_{i}\}=\sum_{i\in \bar{V}}\sum_{j\in V(Z_{i},A_{i})}S_{\beta }(Y_{j}|X_{j})/n^{V}(Z_{i},A_{i})=\sum_{i\in V}\{n^{\bar{V}}(Z_{i},A_{i})/n^{V}(Z_{i},A_{i})\}S_{\beta }(Y_{i}|X_{i}),$$

we have

(A.1) $$\sum_{i=1}^{n}W_{i}^{E1}=\sum_{i\in V}(1+\frac{n^{\bar{V}}(Z_{i},A_{i})}{n^{V}(Z_{i},A_{i})})S_{\beta }(Y_{i}|X_{i})=\sum_{i=1}^{n}\frac{\xi_{i}}{{\hat{\pi }}_{i}^{z}}S_{\beta }(Y_{i}|X_{i})=\sum_{i=1}^{n}W_{i}^{I1}.$$

This shows that the mean score estimator *β̂*_*E*1_ is the same as the IPW estimator *β̂_I_*_1_. Further, since

$$\sum_{i=1}^{n}(1-\frac{\xi_{i}}{{\hat{\pi }}_{i}^{z}})\hat{E}\{S_{\beta }(Y|X_{i})|Z_{i},A_{i}\}=\sum_{i\in \bar{V}}\sum_{j\in V(Z_{i}A_{i})}\frac{S_{\beta }(Y_{j}|X_{j})}{n^{V}(Z_{i},A_{i})}-\sum_{i\in V}\frac{n^{\bar{V}}(Z_{i},A_{i})}{n^{V}(Z_{i},A_{i})}\sum_{j\in V(Z_{i}A_{i})}\frac{S_{\beta }(Y_{j}|X_{j})}{n^{V}(Z_{i},A_{i})}=\sum_{i\in V}\frac{n^{\bar{V}}(Z_{i},A_{i})}{n^{V}(Z_{i},A_{i})}S_{\beta }(Y_{i}|X_{i})-\sum_{i\in V}\frac{n^{\bar{V}}(Z_{i},A_{i})}{n^{V}(Z_{i},A_{i})}S_{\beta }(Y_{i}|X_{i})=0,$$

we have $\sum_{i=1}^{n}W_{i}^{A1}=\sum_{i=1}^{n}W_{i}^{I1}$. Thus the AIPW estimator *β̂_A_*_1_, the IPW estimator *β̂_I_*_1_ and the mean score estimator *β̂_E_*_1_ are equivalent to each other.

Note that

(A.2) $$\sum_{i=1}^{n}(1-\frac{\xi_{i}}{{\hat{\pi }}_{i}^{z}})\hat{E}\{S_{\beta }(Y|X_{i})|X_{i},A_{i}\}\;=\sum_{i\in \bar{V}}\sum_{j\in V(X_{i},A_{i})}\frac{S_{\beta }(Y_{j}|X_{j})}{n^{V}(X_{i},A_{i})}-\sum_{i\in V}\frac{n^{\bar{V}}(Z_{i},A_{i})}{n^{V}(Z_{i},A_{i})}\sum_{j\in V(X_{i},A_{i})}\frac{S_{\beta }(Y_{j}|X_{j})}{n^{V}(X_{i},A_{i})}\;=\sum_{i\in V}\frac{n^{\bar{V}}(X_{i},A_{i})}{n^{V}(X_{i},A_{i})}S_{\beta }(Y_{i}|X_{i})-\sum_{i\in V}\frac{n^{V}(X_{i},A_{i})}{n^{V}(X_{i},A_{i})}\frac{n^{\bar{V}}(Z_{i},A_{i})}{n^{V}(Z_{i},A_{i})}S_{\beta }(Y_{i}|X_{i})\;=\sum_{i\in V}\{\frac{n^{\bar{V}}(X_{i},A_{i})}{n^{V}(X_{i},A_{i})}-\frac{n^{\bar{V}}(Z_{i},A_{i})}{n^{V}(Z_{i},A_{i})}\}S_{\beta }(Y_{i}|X_{i}),$$

which is not zero unless $Z_{i}^{c}$ is linearly related to *Z_i_* and in this case *β* is not identifiable. Hence the AIPW estimator *β̂_A_*_2_ is different from the AIPW estimator *β̂_A_*_1_.

By (A.1) and (A.2), we have

$$\sum_{i=1}^{n}W_{i}^{A2}=\sum_{i\in V}\{\frac{n(Z_{i},A_{i})}{n^{V}(Z_{i},A_{i})}+\frac{n^{\bar{V}}(X_{i},A_{i})}{n^{V}(X_{i},A_{i})}-\frac{n^{\bar{V}}(Z_{i},A_{i})}{n^{V}(Z_{i},A_{i})}\}S_{\beta }(Y_{i}|X_{i})=\sum_{i\in V}\{1+\frac{n^{\bar{V}}(X_{i},A_{i})}{n^{V}(X_{i},A_{i})}\}S_{\beta }(Y_{i}|X_{i})=\sum_{i=1}^{n}W_{i}^{I2}.$$

Following the same arguments leading to (A.1), we also have $\sum_{i=1}^{n}W_{i}^{E2}=\sum_{i=1}^{n}W_{i}^{I2}$. Hence, the estimators *β̂_I_*_2_, *β̂_E_*_2_ and *β̂_A_*_2_ are equivalent. By following the steps in (A.2), we also have $\sum_{i=1}^{n}(1-\frac{\xi_{i}}{{\hat{\pi }}_{i}})\hat{E}\{S_{\beta }(Y|X_{i})|X_{i},A_{i}\}=0$. Hence, *β̂_A_*_3_ is the same as *β̂_I_*_2_. Therefore, these are essentially two different estimators.

### Proof of Theorem 1

Applying the first order Taylor expansion, *π̃_i_* − *π_i_* = (∂*π*(*X_i_*, *A_i_*, *ψ*_0_)/*∂ψ*)′ (*ψ̂*−*ψ*_0_) + *o_p_* (*n*^−1/2^). From (13), we have

(A.3) $$n^{-1/2}U_{I}=n^{-1/2}\sum_{i=1}^{n}\frac{\xi_{i}}{\pi_{i}}S_{\beta }(Y_{i}|X_{i})-n^{-1/2}\sum_{i=1}^{n}\frac{{\tilde{\pi }}_{i}-\pi_{i}}{{\tilde{\pi }}_{i}\pi_{i}}\xi_{i}S_{\beta }(Y_{i}|X_{i})$$

The second term of (A.3) is

(A.4) $$n^{-1/2}\sum_{i=1}^{n}\frac{{\tilde{\pi }}_{i}-\pi_{i}}{{\tilde{\pi }}_{i}\pi_{i}}\xi_{i}S_{\beta }(Y_{i}|X_{i})=n^{-1/2}\sum_{i=1}^{n}\pi_{i}^{-2}\xi_{i}S_{\beta }(Y_{i}|X_{i}){\left(\frac{\partial \pi (X_{i},A_{i},\psi_{0})}{\partial \psi }\right)}^{\prime }(\hat{\psi }-\psi_{0})=E\{\pi_{i}^{-2}\xi_{i}S_{\beta }(Y_{i}|X_{i}){\left(\frac{\partial \pi (X_{i},A_{i},\psi_{0})}{\partial \psi }\right)}^{\prime }\}n^{1/2}(\hat{\psi }-\psi_{0})+o_{p}(1)$$

By (11), (A.3) and (A.4), we have

$$n^{-1/2}U_{I}=n^{-1/2}\sum_{i=1}^{n}(\frac{\xi_{i}}{\pi_{i}}S_{\beta }(Y_{i}|X_{i})-O_{i})+o_{p}(1)=n^{-1/2}\sum_{i=1}^{n}Q_{i}^{I}+o_{p}(1).$$

Now consider the AIPW estimator *β̂_A_* based on solving the estimating equation (14). For simplicity, we denote *E_a_* {*S_β_*(*Y*|*X_i_*)|*X_i_*, *A_i_*} by *E_i_* and *Ẽ* {*S_β_*(*Y*|*X_i_*)|*X_i_*, *A_i_*} by *Ẽ_i_*. We note that

$$n^{-1/2}U_{A}=n^{-1/2}\sum_{i=1}^{n}[\frac{\xi_{i}}{\pi_{i}}S_{\beta }(Y_{i}|X_{i})+(1-\frac{\xi_{i}}{\pi_{i}})E_{i}]+n^{-1/2}\sum_{i=1}^{n}[(\frac{\xi_{i}}{{\tilde{\pi }}_{i}}-\frac{\xi_{i}}{\pi_{i}})\{S_{\beta }(Y_{i}|X_{i})-E_{i}\}+(1-\frac{\xi_{i}}{{\tilde{\pi }}_{i}})({\tilde{E}}_{1}-E_{i})].$$

Suppose that *π̃_i_* and *Ẽ_i_* are the estimates of *π_i_* and *E_i_* based on some parametric or non-parametric models. Then it can be shown using Taylor expansion and standard probability arguments that the second term is at the order of *o_p_*(1) under MAR I. Hence

$$n^{-1/2}U_{A}=n^{-1/2}\sum_{i=1}^{n}[\frac{\xi_{i}}{\pi_{i}}S_{\beta }(Y_{i}|X_{i})+(1-\frac{\xi_{i}}{\pi_{i}})E_{i}]+o_{p}(1).$$

It can be shown that under MAR I, $n^{-1}\partial U_{I}/\partial \beta \overset{P}{\to }I(\beta )$ and $n^{-1}\partial U_{A}/\partial \beta \overset{P}{\to }I(\beta )$. By routine derivations, we have

$$\begin{array}{c} n^{1/2}({\hat{\beta }}_{I}-\beta )=I^{-1}(\beta )n^{-1/2}U_{I}+o_{p}(1)=I^{-1}(\beta )n^{-1/2}\sum_{i=1}^{n}Q_{i}^{I}+o_{p}(1),\\ n^{1/2}({\hat{\beta }}_{A}-\beta )=I^{-1}(\beta )n^{-1/2}U_{A}+o_{p}(1)=I^{-1}(\beta )n^{-1/2}\sum_{i=1}^{n}Q_{i}^{A}+o_{p}(1). \end{array}$$

By the central limit theorem, both *n*^1/2^ (*β̂_I_* − *β*) and *n*^1/2^ (*β̂_A_* − *β*) have asymptotically normal distributions with mean zero and covariances equal to $I^{-1}(\beta )\text{Var}(Q_{i}^{I})I^{-1}(\beta )$ and $I^{-1}(\beta )\text{Var}(Q_{i}^{A})I^{-1}(\beta )$, respectively.

Next, we examine the covariance matrices $\text{Var}(Q_{i}^{I})$ and $\text{Var}(Q_{i}^{A})$ to understand the efficiency gain of *β̂_A_* over *β̂_I_*. Note that $Q_{i}^{I}=\xi_{i}/\pi_{i}S_{\beta }(Y_{i}|X_{i})-O_{i}$ and $Q_{i}^{A}=\xi_{i}/\pi_{i}S_{\beta }(Y_{i}|X_{i})+(1-\xi_{i}/\pi_{i})E_{i}$. Denote *A_i_* = *ξ_ii_*/*π_i_S_β_*(*Y_i_*|*X_i_*) and *B_i_* = (1−*ξ_i_*/*π_ii_*)*E_i_*. Then $Q_{i}^{I}=Q_{i}^{A}-B_{i}-O_{i}$. Under MAR I, $\text{Cov}(Q_{i}^{A},O_{i})=E(Q_{i}^{A}O_{i})=E\{E(Q_{i}^{A}|\xi_{i},X_{i},A_{i})O_{i}\}=E\{E_{i}O_{i}\}=E\{E_{i}E(O_{i}|X_{i},A_{i})\}=0$, and

$$\text{Cov}(Q_{i}^{A},B_{i})=E[\{\frac{\xi_{i}}{\pi_{i}}S_{\beta }(Y_{i}|X_{i})+(1-\frac{\xi_{i}}{\pi_{i}})E_{i}\}(1-\frac{\xi_{i}}{\pi_{i}})E_{i}]=E[{\left(1-\frac{\xi_{i}}{\pi_{i}}\right)}^{2}E_{i}^{2}]-E[{\left(1-\frac{\xi_{i}}{\pi_{i}}\right)}^{2}S_{\beta }(Y_{i}|X_{i})E_{i}]+E[(1-\frac{\xi_{i}}{\pi_{i}})S_{\beta }(Y_{i}|X_{i})E_{i}]=0.$$

Hence, $\text{Cov}(Q_{i}^{A},B_{i}+O_{i})=0$. It follows that $\text{Var}(Q_{i}^{I})=\text{Var}(Q_{i}^{A})+\text{Var}(B_{i}+O_{i})$. Since $Q_{i}^{A}=S_{\beta }(Y_{i}|X_{i})-(1-\frac{\xi_{i}}{\pi_{i}})\{S_{\beta }(Y_{i}|X_{i})-E_{i}\}$ and the two terms are uncorrelated under MAR I, we have $\text{Var}(Q_{i}^{A})=\text{Var}(S_{\beta }(Y_{i}|X_{i}))+\text{Var}((1-\xi_{i}/\pi_{i})\{S_{\beta }(Y_{i}|X_{i})-E_{i}\})$, where the first term equals *I*(*β*). This completes the proof of Theorem 1.

### Proof of Corollary 1

Let $Q_{i}^{A1}=\xi_{i}/\pi_{i}^{z}S_{\beta }(Y_{i}|X_{i})+(1-\xi_{i}/\pi_{i}^{z})E\{S_{\beta }(Y|X_{i})Z_{i},A_{i}\}$, and $Q_{i}^{A2}=\xi_{i}/\pi_{i}^{z}S_{\beta }(Y_{i}|X_{i})+(1-\xi_{i}/\pi_{i}^{z})E\{S_{\beta }(Y|X_{i})|X_{i},A_{i}\}$. By (16),

$$\begin{array}{c} \text{Var}(Q_{i}^{A1})=\text{Var}(S_{\beta }(Y_{i}|X_{i}))+\text{Var}((1-\frac{\xi_{i}}{\pi_{i}^{z}})[S_{\beta }(Y_{i}|X_{i})-E\{S_{\beta }(Y|X_{i})|Z_{i},A_{i}\}]),\\ \text{Var}(Q_{i}^{A2})=\text{Var}(S_{\beta }(Y_{i}|X_{i}))+\text{Var}((1-\frac{\xi_{i}}{\pi_{i}^{z}})[S_{\beta }(Y_{i}|X_{i})-E\{S_{\beta }(Y|X_{i})|X_{i},A_{i}\}]). \end{array}$$

The second term of $\text{Var}(Q_{i}^{A1})$ equals Σ*_A_*_1_(*β*) and the second term of $\text{Var}(Q_{i}^{A2})$ equals Σ*_A_*_2_(*β*). Then it follows from the main results in Theorem 1 that (17) and (18) hold.

Also by Theorem 1, the difference in the variances of $Q_{i}^{A1}$ and $Q_{i}^{A2}$ contributes to the difference in the asymptotic variances of *β̂_A_*_1_ and *β̂_A_*_2_. Since $E\{{\left(1-\xi_{i}/\pi_{i}^{z}\right)}^{2}|Y_{i},X_{i},A_{i}\}=E\{{\left(1-\xi_{i}/\pi_{i}^{z}\right)}^{2}|X_{i},A_{i}\}=(1-\pi_{i}^{z})/\pi_{i}^{z}$ under MAR I,

$$\sum_{A2}(\beta )=E(\frac{1-\pi_{i}^{z}}{\pi_{i}^{z}}\{E\{S_{\beta }^{2}(Y_{i}|X_{i})|X_{i},A_{i}\}-{\left[E\{S_{\beta }(Y|X_{i})|X_{i},A_{i}\}\right]}^{2}\})=E(\frac{1-\pi_{i}^{z}}{\pi_{i}^{z}}\{E\{S_{\beta }^{2}(Y_{i}|X_{i})|Z_{i},A_{i}\}-{\left[E\{S_{\beta }(Y|X_{i})|Z_{i},A_{i}\}\right]}^{2}\})-E(\frac{1-\pi_{i}^{z}}{\pi_{i}^{z}}\{{\left[E\{S_{\beta }(Y|X_{i})|X_{i},A_{i}\}\right]}^{2}-{\left[E\{S_{\beta }(Y|X_{i})|Z_{i},A_{i}\}\right]}^{2}\})=\sum_{A1}(\beta )-E(\frac{1-\pi_{i}^{z}}{\pi_{i}^{z}}\text{Var}([E\{S_{\beta }(Y|X_{i})|X_{i},A_{i}\}]|Z_{i},A_{i})),$$

which is less than Σ*_A_*_1_ (*β*) if the covariates *Z_i_* is a proper subset of *X_i_*.

### Proof of Corollary 2

Consider the definitions of *B_i_* and *O_i_* given following (16). We note that

$$E\{\pi_{i}^{-1}S_{\beta }(Y_{i}|X_{i}){\left(\frac{\partial \pi (Z_{i},A_{i},\psi_{0})}{\partial \psi }\right)}^{\prime }\}=E[\pi_{i}^{-1}E\{S_{\beta }(Y_{i}|X_{i})|Z_{i},A_{i}\}{\left(\frac{\partial \pi (Z_{i},A_{i},\psi_{0})}{\partial \psi }\right)}^{\prime }]=\sum_{z,a}\rho (z,a)\pi^{-1}(z,a,\psi_{0})E\{S_{\beta }(Y|X)|Z=z,A=a\}{\left(\frac{\partial \pi (z,a,\psi_{0})}{\partial \psi }\right)}^{\prime },$$

and

$$n^{-1/2}\sum_{i=1}^{n}S_{i}^{\psi }=n^{-1/2}\sum_{i=1}^{n}\frac{\left(\xi_{i}-\pi (Z_{i},A_{i},\psi_{0})\right)}{\pi (Z_{i},A_{i},\psi_{0})(1-\pi (Z_{i},A_{i},\psi_{0}))}\frac{\partial \pi (Z_{i},A_{i},\psi_{0})}{\partial \psi }=\sum_{z,a}n^{-1/2}\sum_{j=1}^{n}\frac{\left(\xi_{j}-\pi (z,a,\psi_{0}))I(Z_{j}=z,A_{j}=a\right)}{\pi (z,a,\psi_{0})(1-\pi (z,a,\psi_{0}))}\frac{\partial \pi (z,a,\psi_{0})}{\partial \psi }.$$

From the discussions preceding Corollary 2, *ѱ* = {*ѱ*_*z*, *a*_} and *π*(*z*, *a*, *ѱ*) = *ѱ*_*z*, *a*_, where *ѱ*_*z*, *a*_ = *P*(*ξ_i_* = 1|*Z_i_* = *z*, *A_i_* = *a*) for all distinct pairs (*z*, *a*). Hence, $\frac{\partial \pi (z,a,\psi_{0})}{\partial \psi }$ is a column vector with 1 in the position for *ѱ*_*z*, *a*_ and 0 elsewhere. And *I^ѱ^* is a diagonal matrix and its inverse matrix is also diagonal.

We have

$$n^{-1/2}\sum_{i=1}^{n}O_{i}=E\{\pi_{i}^{-1}S_{\beta }(Y_{i}|X_{i}){\left(\frac{\partial \pi (Z_{i},A_{i},\psi_{0})}{\partial \psi }\right)}^{\prime }\}{\left(I^{\psi }\right)}^{-1}n^{-1/2}\sum_{i=1}^{n}S_{i}^{\psi }=\sum_{z,a}E\{S_{\beta }(Y|X)|Z=z,A=a\}n^{-1/2}\sum_{j=1}^{n}\frac{\xi_{j}-\pi (z,a,\psi_{0})}{\pi (z,a,\psi_{0})}I(Z_{j}=z,A_{j}=a),$$

and

$$n^{-1/2}\sum_{i=1}^{n}B_{i}=-n^{-1/2}\sum_{i=1}^{n}\frac{\left(\xi_{i}-\pi (Z_{i},A_{i})\right)}{\pi (Z_{i},A_{i})}E_{i}=-n^{-1/2}\sum_{i=1}^{n}\frac{\left(\xi_{i}-\pi (Z_{i},A_{i})\right)}{\pi (Z_{i},A_{i})}E\{S_{\beta }(Y_{i}|X_{i})|X_{i},A_{i}\}=-\sum_{z,a}n^{-1/2}\sum_{j=1}^{n}\frac{\xi_{j}-\pi (z,a,\psi_{0})}{\pi (z,a,\psi_{0})}I(Z_{j}=z,A_{j}=a)E\{S_{\beta }(Y_{j}|X_{j})|Z_{j}=z,Z_{j}^{c},A_{j}=a\}.$$

Then (21) holds. It follows that *β̂_A_* is more efficient than *β̂_I_* unless *Var*{*S_β_*(*Y_j_*|*X_j_*)|*Z_j_* = *z*, *A_j_* = *a*} = 0 for all (*z*, *a*) for which *P*(*Z_i_* = *z*, *A_i_* = *a*) ≠ 0.

## Appendix B

### Proof of the simplified variance formula (27)

The information matrix *I*(*β*) is a (*k* + 1) × (*k* + 1) symmetric matrix given by

$$I(b_{o},b_{1},\theta )=[\begin{array}{ccccc} \sum_{l,m}q_{lm} & \sum_{m=1}^{k}q_{1m} & q_{11}+q_{01} & \cdots & q_{1r}+q_{0r}\\ \sum_{m=1}^{k}q_{1m} & \sum_{m=1}^{k}q_{1m} & q_{11} & \cdots & q_{1r}\\ q_{11}+q_{01} & q_{11} & q_{11}+q_{01} & \cdots & 0\\ \vdots & \vdots & \vdots & \ddots & \vdots \\ q_{1r}+q_{0r} & q_{1r} & 0 & \cdots & q_{1r}+q_{0r} \end{array}]$$

where *r* = *k* − 1 and *q_lm_* = *E*(*T_i_e^b_lm_^I*{individual *i* in category (*l*, *m*)}). For ease of presentation in the following, we drop the augments (*b*_0_, *b*_1_, *θ*) and use *I* for *I*(*b*_0_, *b*_1_, *θ*). Let *I_ij_* be the cofactor of the (*i*, *j*)th element of *I*.

First we need to find the elements on the second row of the information matrix *I*(*b*_0_, *b*_1_, *θ*). Note that for a matrix *A*, ((*a_ij_*)*_n×n_*)^−1^ = (*A_ji_*)*_n×n_*/|*A*|, where *A_ij_* is the cofactor of *a_ij_* in the matrix *A* and |*A*| is the determinant of *A*. Also note that for a block matrix

$$B=(\begin{array}{cc} B_{11} & B_{12}\\ B_{21} & B_{22} \end{array}),$$

the determinant $\left|B|=|B_{22}||B_{11}-B_{12}B_{22}^{-1}B_{21}\right|$. We have

$$|I|=\prod_{m=1}^{k-1}(q_{1m}+q_{0m})|(\begin{array}{cc} \sum_{lm}q_{lm} & \sum_{m=1}^{k}q_{1m}\\ \sum_{m=1}^{k}q_{1m} & \sum_{m=1}^{k}q_{1m} \end{array})-(\begin{array}{cc} \sum_{l=0}^{1}\sum_{m=1}^{k-1}q_{lm} & \sum_{m=1}^{k-1}q_{1m}\\ \sum_{m=1}^{k-1}q_{1m} & \sum_{m=1}^{k-1}\frac{{\left(q_{1m}\right)}^{2}}{q_{1m}+q_{0m}} \end{array})|=\prod_{m=1}^{k-1}(q_{1m}+q_{0m})|\begin{array}{cc} q_{1k}+q_{0k} & q_{1k}\\ q_{1k} & q_{1k}+\sum_{m=1}^{k-1}\frac{q_{0m}q_{1m}}{q_{1m}+q_{0m}} \end{array}|=\prod_{m=1}^{k}(q_{1m}+q_{0m})\sum_{m=1}^{k}\frac{q_{0m}q_{1m}}{q_{1m}+q_{0m}},$$

and

$$I_{21}=-|\begin{array}{cccc} \sum_{m=1}^{k}q_{1m} & q_{11}+q_{01} & \cdots & q_{1r}+q_{0r}\\ q_{11} & q_{11}+q_{01} & \cdots & 0\\ \vdots & \vdots & \ddots & \vdots \\ q_{1r} & 0 & \cdots & q_{1r}+q_{0r} \end{array}|=-\prod_{m=1}^{k-1}(q_{1m}+q_{0m})[\sum_{m=1}^{k}q_{1m}-\sum_{m=1}^{k-1}q_{1m}]=-q_{1k}\prod_{m=1}^{k-1}(q_{1m}+q_{0m}).$$

Hence, the (2, 1)th element of *I*^−1^ is

$${\left(I^{-1}\right)}_{21}=\frac{I_{21}}{\left|I\right|}=-\frac{q_{1k}}{W(q_{1k}+q_{0k})},$$

where $W=\sum_{m=1}^{k}q_{0m}q_{1m}/(q_{1m}+q_{0m})$.

To calculate (2, 2)th element of *I*^−1^, we have

$$I_{22}=|\begin{array}{cccc} \sum_{l,m}^{k}q_{lm} & q_{11}+q_{01} & \cdots & q_{1r}+q_{0r}\\ q_{11}+q_{01} & q_{11}+q_{01} & \cdots & 0\\ \vdots & \vdots & \ddots & \vdots \\ q_{1r}+q_{0r} & 0 & \cdots & q_{1r}+q_{0r} \end{array}|=\prod_{m=1}^{k-1}(q_{1m}+q_{0m})[\sum_{l,m}q_{lm}-\sum_{l=0}^{1}\sum_{m=1}^{k-1}q_{lm}]=\prod_{m=1}^{k}(q_{1m}+q_{0m}).$$

Hence, the (2, 2)th element of *I*^−1^ is (*I*^−1^)_22_ = *I*_22_/|*I*| = *1*/*W*.

To calculate (2, 3)th element of *I*^−1^, we have

$$I_{23}=-|\begin{array}{ccccc} \sum_{l,m}q_{lm} & \sum_{m=1}^{k}q_{1m} & q_{12}+q_{02} & \cdots & q_{1r}+q_{0r}\\ q_{11}+q_{01} & q_{11} & 0 & \cdots & 0\\ q_{12}+q_{02} & q_{12} & q_{12}+q_{02} & \cdots & 0\\ \vdots & \vdots & \vdots & \ddots & \vdots \\ q_{1r}+q_{0r} & q_{1r} & 0 & \cdots & q_{1r}+q_{0r} \end{array}|=-\prod_{m=2}^{k-1}(q_{1m}+q_{0m})|(\begin{array}{cc} \sum_{lm}q_{lm} & \sum_{m=1}^{k}q_{1m}\\ q_{11}+q_{01} & q_{11} \end{array})-(\begin{array}{cc} \sum_{m=2}^{k-1}(q_{1m}+q_{0m}) & \sum_{m=2}^{k-1}q_{1m}\\ 0 & 0 \end{array})|=-\prod_{m=2}^{k-1}(q_{1m}+q_{0m})(q_{0k}q_{11}-q_{1k}q_{01}).$$

Hence

$${\left(I^{-1}\right)}_{23}=\frac{I_{23}}{\left|I\right|}=-\frac{q_{11}}{W(q_{11}+q_{01})}+\frac{q_{1k}}{W(q_{1k}+q_{0k})}.$$

To calculate (2, 4)th element of *I*^−1^, we have

$$I_{24}=|\begin{array}{cccccc} \sum_{l,m}q_{lm} & \sum_{m=1}^{k}q_{1m} & q_{11}+q_{01} & q_{13}+q_{03} & \cdots & q_{1r}+q_{0r}\\ q_{11}+q_{01} & q_{11} & q_{11}+q_{01} & 0 & \cdots & 0\\ q_{12}+q_{02} & q_{12} & 0 & 0 & \cdots & 0\\ q_{13}+q_{03} & q_{13} & 0 & q_{13}+q_{03} & \cdots & 0\\ \vdots & \vdots & \vdots & \vdots & \ddots & \vdots \\ q_{1r}+q_{0r} & q_{1r} & 0 & 0 & \cdots & q_{1r}+q_{0r} \end{array}|.$$

By switching the 2nd and the 3rd row, we have

$$I_{24}=-n^{k}|\begin{array}{cccccc} \sum_{l,m}q_{lm} & \sum_{m=1}^{k}q_{1m} & q_{11}+q_{01} & q_{13}+q_{03} & \cdots & q_{1r}+q_{0r}\\ q_{12}+q_{02} & q_{12} & 0 & 0 & \cdots & 0\\ q_{11}+q_{01} & q_{11} & q_{11}+q_{01} & 0 & \cdots & 0\\ q_{13}+q_{03} & q_{13} & 0 & q_{13}+q_{03} & \cdots & 0\\ \vdots & \vdots & \vdots & \vdots & \ddots & \vdots \\ q_{1r}+q_{0r} & q_{1r} & 0 & 0 & \cdots & q_{1r}+q_{0r} \end{array}|.$$

Hence

$${\left(I^{-1}\right)}_{24}=\frac{I_{24}}{\left|I\right|}=-\frac{q_{12}}{W(q_{12}+q_{02})}+\frac{q_{1k}}{W(q_{1k}+q_{0k})}.$$

In general, to calculate *I*_2(_*_m_*_+2)_ for *m* = 1, …, *k* − 1, we can obtain a matrix with a (*k* − 2) × (*k* − 2) diagonal right lower block by switching rows of *I*_2(_*_m_*_+2)_ even number of times when *m* is odd and by switching rows odd number of times when *m* is even. Then similar to calculating (*I*^−1^)_24_, we have

$${\left(I^{-1}\right)}_{2(m+2)}=-\frac{q_{1m}}{W(q_{1m}+q_{0m})}+\frac{q_{1k}}{W(q_{1k}+q_{0k})}.$$

For *l*= 1 and 1 ≤ *m* ≤ *k*, the (*i*, *j*)th element of *G_lm_* is *g_ij_* = 1 for (*i*, *j*) = (1, 1), (1, 2), (1, *m* + 2), (2, 1), (2, 2), (2, *m* + 2), (*m* + 2, 1), (*m* + 2, 2), (*m* + 2, *m* + 2), and *g_ij_* = 0 elsewhere. We have the (2, 2)th element of *I*^−1^ *G_lm_I*^−1^ as

$${\left(I^{-1}\right)}_{21}^{2}+{\left(I^{-1}\right)}_{22}^{2}+{\left(I^{-1}\right)}_{2(m+2)}^{2}+2{\left(I^{-1}\right)}_{21}{\left(I^{-1}\right)}_{2(m+2)}+2{\left(I^{-1}\right)}_{22}{\left(I^{-1}\right)}_{2(m+2)}+2{\left(I^{-1}\right)}_{21}{\left(I^{-1}\right)}_{22}=\frac{1}{W^{2}}{\left(\frac{q_{0m}}{q_{1m}+q_{0m}}\right)}^{2}.$$

Since for *l* = 0 and 1 ≤ *m* ≤ *k*, *g_ij_* = 1 for (*i*, *j*) = (1, 1), (1, *m* + 2), (*m* + 2, 1), (*m* + 2, *m* + 2), and *g_ij_* = 0 elsewhere, in this case the (2, 2)th element of *I*^−1^ *G_lm_I*^−1^ is

$${\left(I^{-1}\right)}_{21}^{2}+{\left(I^{-1}\right)}_{2(m+2)}^{2}+2{\left(I^{-1}\right)}_{21}{\left(I^{-1}\right)}_{2(m+2)}={\left({\left(I^{-1}\right)}_{21}+{\left(I^{-1}\right)}_{2(m+2)}\right)}^{2}=\frac{1}{W^{2}}{\left(\frac{q_{1m}}{q_{1m}+q_{0m}}\right)}^{2}.$$

Hence the (2, 2)th element of the asymptotic covariance matrix of *β̂_A_*_2_ is given by $\sigma_{b_{1}}^{2}=1/W+U/W^{2}$, where

$$U=\sum_{\alpha ,l,m}\rho (\alpha ,l,m)\frac{1-\rho^{V}(\alpha ,l,m)}{\rho^{V}(\alpha ,l,m)}\alpha p_{lm}(1-p_{lm}){\left(\frac{q_{(1-l)m}}{q_{0m}+q_{1m}}\right)}^{2}.$$

Note that *P*(*Y_i_* = 1|*A_i_* = 1, *i* ∈ category (*l*, *m*)) can be estimated by $y_{lm}/n_{lm}^{v}$ and *ρ*(*l*, *m*) by *α_lm_*/*n*. Thus *q_lm_* can be estimated by (*α_lm_*/*n*) $\left(y_{lm}/n_{lm}^{v}\right)$. Then we can estimate *W* by $\hat{W}=n^{-1}\sum_{m=1}^{k}\alpha_{0m}\alpha_{1m}y_{0m}y_{1m}/(\alpha_{0m}y_{0m}n_{1m}^{v}+\alpha_{1m}y_{1m}n_{0m}^{v})$. By replacing $\rho_{l,m}^{v}$ with $n_{lm}^{v}/\alpha_{lm}$ and *p_lm_* with $y_{lm}/n_{lm}^{v}$, we can estimate *U* by

$$\hat{U}=n^{-1}\sum_{l,m}\alpha_{lm}\frac{\alpha_{lm}-n_{lm}^{v}}{n_{lm}^{v}}\frac{y_{lm}}{n_{lm}^{v}}\frac{n_{lm}^{v}-y_{lm}}{n_{lm}^{v}}(\alpha_{(1-l)m}\frac{y_{(1-l)m}}{n_{(1-l)m}^{v}}){\left(\alpha_{1m}\frac{y_{1m}}{n_{1m}^{v}}+\alpha_{0m}\frac{y_{0m}}{n_{0m}^{v}}\right)}^{-2}=n^{-1}\sum_{m=1}^{k}[\frac{\alpha_{1m}-n_{1m}^{v}}{n_{1m}^{v}}\frac{n_{1m}^{v}-y_{1m}}{n_{1m}^{v}}\alpha_{0m}\frac{y_{0m}}{n_{0m}^{v}}+\alpha_{0m}-n_{0m}^{v}n_{0m}^{v}\frac{n_{0m}^{v}-y_{0m}}{n_{0m}^{v}}\alpha_{1m}\frac{y_{1m}}{n_{1m}^{v}}]\times (\alpha_{1m}\frac{y_{1m}}{n_{1m}^{v}}\alpha_{0m}\frac{y_{0m}}{n_{0m}^{v}}){\left(\alpha_{1m}\frac{y_{1m}}{n_{1m}^{v}}+\alpha_{0m}\frac{y_{0m}}{n_{0m}^{v}}\right)}^{-2}=n^{-1}\sum_{m=1}^{k}[(\frac{1}{y_{1m}}-\frac{1}{n_{1m}^{v}}+\frac{1}{\alpha_{1m}}-\frac{n_{1m}^{v}}{\alpha_{1m}y_{1m}})+(\frac{1}{y_{0m}}-\frac{1}{n_{0m}^{v}}+\frac{1}{\alpha_{0m}}-\frac{n_{0m}^{v}}{\alpha_{0m}y_{0m}})]\times {\left(\frac{\alpha_{1m}y_{1m}\alpha_{0m}y_{0m}}{\alpha_{1m}y_{1m}n_{0m}^{v}+\alpha_{0m}y_{0m}n_{1m}^{v}}\right)}^{2}.$$

From *Ŵ* and *Û*, we obtain an estimate of $\sigma_{b_{1}}^{2}$ as follows

$${\hat{\sigma }}_{b_{1}}^{2}=\frac{1}{\hat{W}}\sum_{m=1}^{k}[(\frac{1}{y_{1m}}-\frac{1}{n_{1m}^{v}}+\frac{1}{\alpha_{1m}})+(\frac{1}{y_{0m}}-\frac{1}{n_{0m}^{v}}+\frac{1}{\alpha_{0m}})]{\left(\frac{\alpha_{1m}y_{1m}\alpha_{0m}y_{0m}}{\alpha_{1m}y_{1m}n_{0m}^{v}+\alpha_{0m}y_{0m}n_{1m}^{v}}\right)}^{2}+\frac{1}{\hat{W}}+\frac{1}{{\hat{W}}^{2}}\sum_{m=1}^{k}[-\frac{n_{1m}^{v}}{\alpha_{1m}y_{1m}}-\frac{n_{0m}^{v}}{\alpha_{0m}y_{0m}}]{\left(\frac{\alpha_{1m}y_{1m}\alpha_{0m}y_{0m}}{\alpha_{1m}y_{1m}n_{0m}^{v}+\alpha_{0m}y_{0m}n_{1m}^{v}}\right)}^{2}=\frac{1}{{\hat{W}}^{2}}\sum_{m=1}^{k}[(\frac{1}{y_{1m}}-\frac{1}{n_{1m}^{v}}+\frac{1}{\alpha_{1m}}+\frac{1}{y_{0m}}-\frac{1}{n_{0m}^{v}}+\frac{1}{\alpha_{0m}})]{\left(\frac{\alpha_{1m}y_{1m}\alpha_{0m}y_{0m}}{\alpha_{1m}y_{1m}n_{0m}^{v}+\alpha_{0m}y_{0m}n_{1m}^{v}}\right)}^{2}.$$

The variance of *b̂*_1_ is estimated by $\hat{\text{Var}}({\hat{b}}_{1})=n^{-1}{\hat{\sigma }}_{b_{1}}^{2}$.

## References

[R1] Carpenter JR, Kenward MG, Vansteelandt S (2006). A comparison of multiple imputation and doubly robust estimation
for analyses with missing data. J R Stat Soc A.

[R2] Clayton D, Spiegelhalter D, Dunn G, Pickles A (1998). Analysis of longitudinal binary data from multiphase sampling
(with discussion). J R Stat Soc B.

[R3] Chu H, Halloran EM (2004). Estimating vaccine efficacy using auxiliary outcome data and a
small validation sample. Stat Med.

[R4] Dempster AP, Laird NM, Rubin DB (1977). Maximum likelihood from incomplete data via the EM
algorithm. J R Stat Soc B.

[R5] Ellenberg SS, Hamilton JM (1989). Surrogate endpoints in clinical trials: cancer. Stat Med.

[R6] Fleming TR (1992). Evaluating therapeutic interventions: some issues and
experiences. Stat Sci.

[R7] Halloran EM, Longini IM, Gaglani MJ, Piedra PA, Chu H, Herschler GB, Glezed WP (2003). Estimating efficacy of trivalent, cold-adapted, influenza virus
vaccine (CAIV-T) against influenza A (H1N1) and B Using Surveillance
Cultures. Am J Epid.

[R8] Horvitz DG, Thompson DJ (1952). A generalization of sampling without replacement from a finite
universe. J Am Stat Assoc.

[R9] Little RJA, Rubin DB (2002). Statistical analysis of missing data.

[R10] Pepe MS, Reilly M, Fleming TR (1994). Auxiliary outcome data and the mean score method. J Stat Plan Inference.

[R11] Prentice RL (1989). Surrogate endpoints in clinical trials: definition and
operational criteria. Stat Med.

[R12] Rubin DB (1987). Multiple imputation for nonresponse in surveys.

[R13] Robins JM, Rotnitzky A, Zhao LP (1994). Estimation of regression coefficients when some regressors are
not always observed. J Am Stat Assoc.

[R14] Scharfstein DO, Rotnitzky A, Robins JM (1999). Adjusting for nonignorable drop-out using semiparametric
nonresponse models: rejoinder. J Am Stat Assoc.

